# Fibroblast exosomes promote wound healing and improve the quality of healed skin via miR-29a-3p-mediated KEAP1/Nrf2 pathway activation

**DOI:** 10.1093/burnst/tkaf035

**Published:** 2025-05-17

**Authors:** Lingfeng Yan, Dejiang Fan, Jiacai Yang, Jue Wang, Xiaohong Hu, Xiaorong Zhang, Yong Huang, Hong Wang, Wenjing Yin, Xin Cai, Ruoyu Shang, Canhua Huang, Gaoxing Luo, Weifeng He

**Affiliations:** Institute of Burn Research, State Key Laboratory of Trauma and Chemical Poisoning, the First Affiliated Hospital of Army Medical University (the Third Military Medical University), 30 Gaotanyan Main Street, Shapingba District, Chongqing 400038, China; Chongqing Key Laboratory for Wound Repair and Tissue Regeneration, Army Medical University (Third Military Medical University), 30 Gaotanyan Main Street, Shapingba District, Chongqing 400000, China; Institute of Burn Research, State Key Laboratory of Trauma and Chemical Poisoning, the First Affiliated Hospital of Army Medical University (the Third Military Medical University), 30 Gaotanyan Main Street, Shapingba District, Chongqing 400038, China; Chongqing Key Laboratory for Wound Repair and Tissue Regeneration, Army Medical University (Third Military Medical University), 30 Gaotanyan Main Street, Shapingba District, Chongqing 400000, China; Institute of Burn Research, State Key Laboratory of Trauma and Chemical Poisoning, the First Affiliated Hospital of Army Medical University (the Third Military Medical University), 30 Gaotanyan Main Street, Shapingba District, Chongqing 400038, China; Chongqing Key Laboratory for Wound Repair and Tissue Regeneration, Army Medical University (Third Military Medical University), 30 Gaotanyan Main Street, Shapingba District, Chongqing 400000, China; Institute of Burn Research, State Key Laboratory of Trauma and Chemical Poisoning, the First Affiliated Hospital of Army Medical University (the Third Military Medical University), 30 Gaotanyan Main Street, Shapingba District, Chongqing 400038, China; Chongqing Key Laboratory for Wound Repair and Tissue Regeneration, Army Medical University (Third Military Medical University), 30 Gaotanyan Main Street, Shapingba District, Chongqing 400000, China; Institute of Burn Research, State Key Laboratory of Trauma and Chemical Poisoning, the First Affiliated Hospital of Army Medical University (the Third Military Medical University), 30 Gaotanyan Main Street, Shapingba District, Chongqing 400038, China; Chongqing Key Laboratory for Wound Repair and Tissue Regeneration, Army Medical University (Third Military Medical University), 30 Gaotanyan Main Street, Shapingba District, Chongqing 400000, China; Institute of Burn Research, State Key Laboratory of Trauma and Chemical Poisoning, the First Affiliated Hospital of Army Medical University (the Third Military Medical University), 30 Gaotanyan Main Street, Shapingba District, Chongqing 400038, China; Chongqing Key Laboratory for Wound Repair and Tissue Regeneration, Army Medical University (Third Military Medical University), 30 Gaotanyan Main Street, Shapingba District, Chongqing 400000, China; Institute of Burn Research, State Key Laboratory of Trauma and Chemical Poisoning, the First Affiliated Hospital of Army Medical University (the Third Military Medical University), 30 Gaotanyan Main Street, Shapingba District, Chongqing 400038, China; Chongqing Key Laboratory for Wound Repair and Tissue Regeneration, Army Medical University (Third Military Medical University), 30 Gaotanyan Main Street, Shapingba District, Chongqing 400000, China; Institute of Burn Research, State Key Laboratory of Trauma and Chemical Poisoning, the First Affiliated Hospital of Army Medical University (the Third Military Medical University), 30 Gaotanyan Main Street, Shapingba District, Chongqing 400038, China; Chongqing Key Laboratory for Wound Repair and Tissue Regeneration, Army Medical University (Third Military Medical University), 30 Gaotanyan Main Street, Shapingba District, Chongqing 400000, China; Institute of Burn Research, State Key Laboratory of Trauma and Chemical Poisoning, the First Affiliated Hospital of Army Medical University (the Third Military Medical University), 30 Gaotanyan Main Street, Shapingba District, Chongqing 400038, China; Chongqing Key Laboratory for Wound Repair and Tissue Regeneration, Army Medical University (Third Military Medical University), 30 Gaotanyan Main Street, Shapingba District, Chongqing 400000, China; Institute of Burn Research, State Key Laboratory of Trauma and Chemical Poisoning, the First Affiliated Hospital of Army Medical University (the Third Military Medical University), 30 Gaotanyan Main Street, Shapingba District, Chongqing 400038, China; Chongqing Key Laboratory for Wound Repair and Tissue Regeneration, Army Medical University (Third Military Medical University), 30 Gaotanyan Main Street, Shapingba District, Chongqing 400000, China; Institute of Burn Research, State Key Laboratory of Trauma and Chemical Poisoning, the First Affiliated Hospital of Army Medical University (the Third Military Medical University), 30 Gaotanyan Main Street, Shapingba District, Chongqing 400038, China; Chongqing Key Laboratory for Wound Repair and Tissue Regeneration, Army Medical University (Third Military Medical University), 30 Gaotanyan Main Street, Shapingba District, Chongqing 400000, China; State Key Laboratory of Biotherapy and Cancer Center, West China Hospital, and West China School of Basic Medical Sciences & Forensic Medicine, Sichuan University, and Collaborative Innovation Center for Biotherapy, No. 37 Guoxue Alley, Wuhou District, Chengdu, Sichuan 610041, China; Institute of Burn Research, State Key Laboratory of Trauma and Chemical Poisoning, the First Affiliated Hospital of Army Medical University (the Third Military Medical University), 30 Gaotanyan Main Street, Shapingba District, Chongqing 400038, China; Chongqing Key Laboratory for Wound Repair and Tissue Regeneration, Army Medical University (Third Military Medical University), 30 Gaotanyan Main Street, Shapingba District, Chongqing 400000, China; Institute of Burn Research, State Key Laboratory of Trauma and Chemical Poisoning, the First Affiliated Hospital of Army Medical University (the Third Military Medical University), 30 Gaotanyan Main Street, Shapingba District, Chongqing 400038, China; Chongqing Key Laboratory for Wound Repair and Tissue Regeneration, Army Medical University (Third Military Medical University), 30 Gaotanyan Main Street, Shapingba District, Chongqing 400000, China

**Keywords:** Fibroblast exosomes, Wound healing, Biomechanical integrity, Epidermal barrier, KEAP1/Nrf2 pathway

## Abstract

**Background:**

Wound healing is a sophisticated biological process characterized by the orchestrated interplay of diverse cellular components, growth factors, and signaling cascades. Recent research has highlighted the pivotal role of fibroblast exosomes in mediating intercellular communication and facilitating tissue regeneration. This investigation aimed to elucidate the therapeutic efficacy of fibroblast exosomes in enhancing wound repair mechanisms, with a particular emphasis on their differential effects in normal and diabetic wound healing paradigms.

**Methods:**

A mouse full-thickness skin defect model was used to evaluate the effects of fibroblast exosomes on wound re-epithelialization, granulation tissue formation, and epidermal barrier function. Molecular and cellular experiments were conducted to analyze the roles of exosomes in epidermal stem cell proliferation, migration, differentiation, and antioxidant stress, with further validation of the associated signaling pathways. The therapeutic efficacy was additionally confirmed in a type 1 diabetic mouse model.

**Results:**

Fibroblast exosomes significantly enhanced wound re-epithelialization by promoting the proliferation, migration, and differentiation of epidermal stem cells. Additionally, exosomes increased fibroblast abundance and myofibroblast activation, facilitating granulation tissue formation as well as improving extracellular matrix (ECM) deposition and the biomechanical properties of healed skin. Furthermore, exosomes improved epidermal barrier function by upregulating tight junction proteins (e.g. Claudin-1 and ZO-1) and reducing transepidermal water loss (TEWL). In diabetic mouse models, exosomes accelerated wound closure, restored ECM deposition and biomechanical integrity, and repaired epidermal barrier function. Mechanistically, exosomes target the 3′ untranslated region (UTR) of Keap1 mRNA through miR-29a-3p and activate the KEAP1/Nrf2 antioxidant pathway, mitigating oxidative stress and protecting epidermal stem cells from reactive oxygen species (ROS)-induced damage.

**Conclusion:**

Fibroblast exosomes alleviate oxidative damage by modulating the KEAP1/Nrf2 pathway through miR-29a-3p and enhancing epidermal stem cell function. These exosomes exhibit remarkable therapeutic potential in accelerating wound healing and improving healing quality under both normal and diabetic conditions, offering a robust foundation for innovative therapeutic strategies.

## Background

Wound repair is a sophisticated and dynamic biological process characterized by the orchestrated interaction of diverse cell populations, growth factors, and signaling pathways aimed at restoring the structural integrity and functional capacity of the skin following injury [1]. Despite significant advancements in our understanding of the molecular and cellular mechanisms underlying wound healing, chronic wounds remain a major clinical challenge, affecting millions of individuals worldwide and imposing a substantial burden on health care systems [2,3]. Chronic wounds, such as diabetic foot ulcers, pressure ulcers, and venous leg ulcers, are characterized by delayed or impaired healing, often resulting in persistent inflammation, infection, and tissue damage [4]. With the increasing prevalence of conditions such as diabetes and obesity as well as an aging population, the incidence of chronic wounds is anticipated to increase, underscoring the urgent need for effective therapeutic strategies to enhance wound healing and improve patient outcomes [5].

Exosomes have emerged as promising therapeutic agents for wound healing because of their ability to deliver a wide range of bioactive molecules, such as proteins, lipids, and nucleic acids, to target cells, thereby modulating their function and promoting tissue repair [6]. Exosomes are small extracellular vesicles (30–150 nm in diameter) that are secreted by various cell types and play pivotal roles in intercellular communication [7]. The content and functionality of exosomes are determined by their cell of origin, and exosomes derived from different cell types have been demonstrated to exert distinct effects on wound healing [8]. For example, exosomes derived from mesenchymal stem cells (MSCs) have been extensively studied for their pro-regenerative properties and have been shown to promote angiogenesis, collagen deposition, and immunomodulation in various wound healing models [9]. Similarly, exosomes from adipose-derived stem cells (ASCs) and keratinocytes also have beneficial effects on wound healing, such as enhancing re-epithelialization and reducing inflammation [10].

Fibroblasts are the primary cell type in the dermis and are responsible for the synthesis and remodeling of the extracellular matrix (ECM), rendering them essential for the wound healing process [11,12]. In addition to their role in ECM production, fibroblasts secrete a variety of growth factors, cytokines, and other signaling molecules that regulate the functionality of other cell types, such as keratinocytes, endothelial cells, and immune cells [13–16]. Dysfunction or depletion of fibroblasts has been implicated in the pathogenesis of chronic wounds, underscoring the critical importance of these cells in wound healing [17]. Recent studies have demonstrated that exosomes derived from fibroblasts can promote the proliferation and migration of skin cells in vitro, suggesting their potential therapeutic value in wound repair [18–21]. However, the *in vivo* effects of fibroblast exosomes on wound healing and the specific cellular and molecular mechanisms involved remain largely unexplored.

Epidermal stem cells represent another critical cell type involved in wound healing; they are responsible for the regeneration of the epidermis and restoration of skin barrier function following injury [22,23]. Residing in the basal layer of the epidermis, epidermal stem cells give rise to transit-amplifying cells, which subsequently differentiate into the various cell types that constitute the stratified epidermis [24]. Previous studies have shown that epidermal stem cells are essential for wound re-epithelialization and that their depletion or dysfunction can impair wound closure and lead to chronic wounds [25]. Therefore, strategies that promote the proliferation, migration, and differentiation of epidermal stem cells may enhance wound healing and improve the quality of healed skin. Although several studies have investigated the effects of exosomes derived from other cell types on epidermal stem cell function, the potential impact of fibroblast-derived exosomes on these cells in the context of wound healing remains to be fully explored [26,27].

Oxidative stress has been identified as a major factor contributing to the impairment of wound healing and development of chronic wounds [28]. Elevated levels of reactive oxygen species (ROS) can damage cellular components, impair cell function, and disrupt the delicate balance between tissue repair and destruction [29]. In chronic wounds, persistent inflammation and the overproduction of ROS can lead to the degradation of growth factors and ECM components, further exacerbating impaired wound healing [30]. Moreover, oxidative stress has been shown to negatively impact the function of various cell types involved in wound healing, including fibroblasts, keratinocytes, and endothelial cells [31]. Therefore, strategies that reduce oxidative stress and enhance antioxidant defenses may promote wound healing and improve the quality of healed skin. Recent studies have demonstrated that exosomes derived from various cell types can exert antioxidant effects and protect against oxidative damage [32], highlighting their potential therapeutic value for chronic wounds. However, the antioxidant effects of fibroblast exosomes in the context of wound healing have not been previously investigated.

Diabetes mellitus represents a significant risk factor for the development of chronic wounds, with diabetic foot ulcers affecting up to 25% of individuals with diabetes during their lifetime [33]. Diabetic wounds are characterized by impaired re-epithelialization, reduced angiogenesis, and persistent inflammation, leading to delayed healing and an increased risk of amputation [34]. The pathogenesis of diabetic wounds is multifactorial and involves a complex interplay of hyperglycemia, neuropathy, ischemia, and infection [35]. At the cellular level, diabetes has been shown to impair the function of various cell types involved in wound healing, including fibroblasts, keratinocytes, and endothelial cells, through mechanisms such as oxidative stress, advanced glycation end products (AGEs), and inflammatory cytokines [36]. Consequently, strategies targeting multiple aspects of diabetic wound pathogenesis and enhancing the function of key cell types involved in wound healing may improve outcomes in this challenging patient population. Although several studies have investigated the effects of exosomes derived from other cell types on diabetic wound healing, the potential therapeutic value of fibroblast-derived exosomes in this context remains to be fully explored [37].

In this study, we provide a comprehensive analysis of the effects of fibroblast exosomes on wound healing in both normal and diabetic mice. By combining in vitro and *in vivo* approaches, we demonstrate that fibroblast exosomes promote re-epithelialization by stimulating the proliferation, migration, and differentiation of epidermal stem cells. We also show that fibroblast exosomes enhance granulation tissue formation by increasing fibroblast abundance and myofibroblast activation, leading to increased ECM deposition and improved biomechanical properties of healed skin. Furthermore, we provide evidence that fibroblast-derived exosomes reduce oxidative stress in the wound microenvironment by activating the KEAP1/Nrf2 antioxidant pathway through the action of miR-29a-3p, which targets the 3′UTR. This mechanism protects epidermal stem cells from oxidative damage and enhances their functionality. In conclusion, our findings demonstrate that fibroblast-derived exosomes represent a promising therapeutic strategy for promoting wound healing and improving the quality of healed skin under both normal and diabetic conditions.

## Methods

**Table 1:** Key resources table

Reagent or resource		Source	Identifier
Antibodies			
Anti-Cytokeratin 14 antibody		Abcam	Cat. No. Ab119695
Anti-Cytokeratin 10 antibody		Abcam	Cat. No. Ab76318
Anti-Vimentin antibody		Abcam	Cat. No. Ab92547
Anti-α-SMA antibody		Abcam	Cat. No. Ab7817
Anti-CollagenIantibody		Abcam	Cat. No. Ab138492
Anti-Collagen III antibody		Abcam	Cat. No. Ab6320
Anti-Claudin-1 antibody		Abcam	Cat. No. Ab307692
Anti-CD63 antibody		Abcam	Cat. No. Ab134045
Anti-TSG101 antibody		Abcam	Cat. No. Ab125011
Anti-Alix antibody		Abcam	Cat. No. Ab275377
Anti-ZO-1 antibody		Proteintech	Cat. No. 21773–1-AP
Anti-KEAP1 antibody		HuaBio	Cat. No. HA721525
Anti-Nrf2 antibody		HuaBio	Cat. No. HA721432
iFluor™ 647 Conjugated Anti- Cytokeratin 10 antibody		HuaBio	Cat. No. HA720146F
iFluor™ 555 Conjugated Anti- Cytokeratin 14 antibody		HuaBio	Cat. No. HA720135F
Chemicals, peptides, and recombinant proteins			
ML385		Selleck	Cat. No. S8790
KEAP1		TargetMol	Cat. No. TMPY-03432
Experimental models: Cell lines			
Immortalization of human dermal fibroblasts, HSF		iCell	Cat. No. iCell-0051a
Primer sequences			
*KEAP1*-F	GCACAACTGTATCTATGCTGCTG	Tsingke	
*KEAP1*-R	CCAGGAACGTGTGACCATCATAG	Tsingke	
miR-29a-3p-RT	GTCGTATCGACTGCAGGGTCCGAGGTATTCGCAGTCGATACGACTAACCG	Tsingke	
miR-29a-3p-F	CGGCTAGCACCATCTGAAAT	Tsingke	
common-R	ACTGCAGGGTCCGAGGTATT	Tsingke	
Experimental models: Organisms/strains			
C57BL/6 mice		Army Medical University (the Third Military Medical University	

### Primary murine dermal fibroblast isolation

Primary dermal fibroblasts were isolated from the skin of newborn (0–2 days old) C57BL/6 mice. Briefly, mouse skin was harvested with sterile dissecting scissors and immersed in 0.3% trypsin/GNK solution (HyClone) for 2 h to separate the epidermis and dermis [38]. The dermis was then minced into 1–2-mm pieces and digested with 0.25% trypsin for 15 min. The mixed liquids were centrifuged at 500 × g for 5 min and resuspended in 10% fetal bovine serum DMEM. Our laboratory has previously identified and published articles on fibroblasts [39].

### Primary human epidermal stem cell isolation and culture

Primary epidermal stem cells were isolated from human foreskin tissue [40]. Briefly, the subcutaneous tissue was removed with sterile dissecting scissors, and the tissue was soaked in a 0.3% trypsin/GNK solution (HyClone) at 4°C overnight to separate the dermis and epidermis layers. The epidermis layer was subsequently cut into pieces and digested with 0.25% trypsin for 15 min. The mixture was centrifuged at 500 × g for 5 min, and the pellet was resuspended in EpiLife medium and allowed to adhere to a culture flask coated with IV collagen for 10 min. The nonadherent cells were removed, and an appropriate amount of EpiLife medium was added. The cells were cultured in a constant-temperature incubator (37°C, 5% CO₂, normoxia), and the medium was changed every other day. Our laboratory has previously identified and published papers on epidermal stem cells [41].

### Mouse and human fibroblast exosome isolation

The exosome separation procedures were performed according to the previous experimental procedures of our laboratory [42]. First, fibroblasts (human skin fibroblasts were used for in vitro experiments, and primary mouse fibroblasts were used for *in vivo* experiments) were incubated with exosome-free medium for 48 h, after which the cell culture supernatant was collected. The supernatant was centrifuged at 500 × g for 5 min and 3400 × g for 15 min to remove debris and apoptotic bodies, respectively. Then, the mixture was centrifuged at 10000 × g for 45 min to remove microvesicles. The supernatant was subsequently resuspended and centrifuged at 100000 × g for 70 min to collect the exosomes. The exosomes were washed with an ice-cold PBS and centrifuged at 100000 × g for 70 min to remove contaminating proteins. In accordance with the MISEV2023 guidelines, we characterized the isolated exosomes using protein markers (CD63, TSG101, Alix, and GAPDH), transmission electron microscopy (TEM), and nanoparticle size analysis. Our findings demonstrated that epidermal stem cells can internalize fibroblast exosomes [20, 43, 44] (Figure S7).

### Immunofluorescence

For the immunofluorescence coculture model, cell slides (Solarbio, YA0350) were placed in a 24-well plate and coated with IV collagen solution overnight. For the fibroblast exosome stimulation experiment, 1 × 10^5^ epidermal stem cells were seeded on each cell slide. After 24 h, the cell slides were incubated with primary and secondary antibodies [14]. The antibodies against cytokeratin 10, cytokeratin 14, Claudin-1, ZO-1, and Nrf2 were used. The results were analyzed via the Olympus Image Viewer and Image-Pro Plus 6.0 software.

### Transepithelial electrical resistance (TEER)

Primary epidermal stem cells were cultured in the upper Transwell chamber until fully confluent. The baseline transepithelial electrical resistance was measured before fibroblast exosome treatment. The transepithelial electrical resistance was measured again 24 h after fibroblast exosome treatment. The product of the measured resistance value and the area of the Transwell chamber was the TEER value of the cell, and the difference between the TEER values before and after treatment was used for statistical analysis [45,  46].

### Proliferation analysis

Cell proliferation was measured using a CCK8 cell counting kit (Dojindo, Japan) according to the manufacturer’s recommendations. 5-Ethynyl-2′-deoxyuridine (EdU) staining was performed using an EdU kit (Beyotime, China). Briefly, the cells were fixed, permeabilized, blocked, and stained with a DAPI solution containing an anti-fluorescence quencher (Beyotime, China). The fluorescence signal of the epidermal stem cells was measured under a microscope.

### Scratch wound migration assay

For the scratch wound migration assay, an artificial wound was created in a monolayer of cells using a 10 μl plastic pipette tip. The closure of the scratch was photographed under a microscope at 0, 24, 48, and 72 h. Each experiment was repeated at least 3 times.

### Apoptosis analysis

For cell apoptosis analysis, cells were seeded in 6-well plates at a density of 5 × 10^5^ cells per well, incubated overnight, and then treated with or without H₂O₂ and fibroblast exosomes. An Annexin V apoptosis detection kit (Invitrogen，America) was used, and the degree of cell apoptosis was detected by flow cytometry.

### Animals and excisional wounds

Male sc57bl/6 mice (6–8 weeks old) were purchased from the Animal Research Institute of the Army Military Medical University (Chongqing, China). To induce type 1 diabetes in C57BL/6 mice, streptozotocin (100 mg/kg, S6089, Macklin) was injected intraperitoneally for 3 consecutive days. Nondiabetic mice were given an equal volume of sodium citrate buffer. After 3 days of continuous STZ injection, the mice were allowed to acclimate for 5 days. The blood glucose level was monitored with a glucometer on Day 8 after the first injection of STZ. Mice with a blood glucose level between 16.7 and 33.3 mmol/L were considered to have type 1 diabetes [47]. The type 2 diabetes mouse model was established through a combination of STZ administration and high-fat/high-sucrose diet feeding. Specifically, the mice were continuously fed a high-fat/high-sucrose diet for 4 weeks, followed by intraperitoneal injection of low-dose STZ (50 mg/kg/day) for 5 consecutive days. After STZ treatment, the mice were maintained on the HFSD for an additional 2 weeks with regular blood glucose monitoring. Animals exhibiting sustained hyperglycemia (random blood glucose levels between 16.7 and 33.3 mmol/L) were considered successfully established type 2 diabetes models [48]. Full-thickness 6-mm dermal biopsy perforations were made symmetrically on the shaved back skin. For C57BL/6 mice, GW4869 (S7609, Selleck) was dissolved in DMSO at a concentration of 1.73 mM as a stock solution, and the stock solution was diluted in PBS at a concentration of 0.1 μg/μl for the experiment. After injury, 50 μl of the drug vehicle (0.1% DMSO), GW4869, or GW4869 + fibroblast-derived exosomes (4 μg/μl, a total of 200 μg) was injected locally every other day. For diabetic mice, 50 μl of the drug vehicle (PBS) or fibroblast-derived exosomes (4 μl/μl, a total of 200 μg) was injected locally every other day. Photos were taken on days 0, 3, 7, and 10 post-injury. The wound area was measured using Image-Pro Plus 6.0 software. All the mice were housed in a specific pathogen-free environment at the Animal Research Institute of the Third Military Medical University (Chongqing, China), and all the experiments were carried out in accordance with the protocol approved by the Animal Ethics Committee of the Third Military Medical University. The animal license number is AMUWEC20242076.

### Flow cytometry

Briefly, the dissected wound tissue with a 2-mm edge was cut into small pieces and cultured with 0.1% collagenase I (A004194, Diamond) and 0.01% DNase I (10 104 159 001, Roche). The pieces were subsequently shaken at 37°C until they were digested into single cells. Single cells were subsequently screened through a 70 μm filter. For surface staining, in the single-cell population, the coexpression of Vimentin and α-SMA was used to mark the myofibroblasts in the wound cells. The plasma membranes of live cells and exosomes were directly labeled with DID and DIO dyes, respectively. The stained cells were detected with an Attune Acoustic Focusing Cytometer (Life Technologies, Carlsbad, CA) and analyzed using Flowlo software, version 10.0 (Tree Star, Ashland, OR).

### Tissue histology analysis

Wound tissues were fixed and embedded in 4% paraformaldehyde and paraffin. H&E staining was performed as described previously. For immunohistochemistry and immunofluorescence staining, the samples were dewaxed, hydrated, and incubated with primary antibodies at 4°C overnight. Then, the samples were incubated with secondary antibodies (SP-9001/9002, ZSGB-BIO, Beijing, China) at room temperature for 1 h and finally visualized using DAB staining or fluorescence labeling, followed by microscopic observation and imaging.

### Western blotting

Wound tissues and cells were dissolved in RIPA buffer and mixed with protease/phosphatase inhibitors and phenylmethylsulfonyl fluoride (Beyotime) on ice. Small pieces of wound tissue were lysed with 5 mm steel bead tissue cell inactivator (ABclonal), and the supernatant was collected after centrifugation. The concentration of the collected protein was determined using a BCA kit (Thermo Fisher Scientific). Proteins were separated by SDS–PAGE and transferred onto a 0.22 μm polyvinylidene fluoride membrane (Millipore). The membrane was blocked with 5% skim milk for 1 h and incubated with the primary antibody at 4°C overnight. Finally, the polyvinylidene fluoride membrane was incubated with a horseradish peroxidase-conjugated secondary antibody (1:3000, Beyotime) for 1 h. An enhanced chemiluminescence (ECL) substrate kit (Advanta, 150 301) was used for visualization of the protein bands.

### Determination of reactive oxygen species, lipid oxidation, and glutathione

Cells and wound tissues were collected at 7 days and 28 days. Lipid oxidation (MDA) and total glutathione (GSH) levels were determined using commercial kits according to the manufacturer’s protocols. The MDA level was determined using a lipid oxidation MDA determination kit (Beyotime, Jiangsu, China). The levels of GSH and GSSG were detected using GSH and GSSG detection kits (Beyotime, China). The frozen wound was stained with dihydroethamine (DHE) (Invitrogen) to detect the generation of ROS *in situ* in the liver, and its oxidation produced a fluorescent derivative, ethylamine.

### Scanning electron microscopy

Twenty-eight-day-old wound tissues were cut and immersed in a 37°C GNK solution for 4 h, after which the epidermal tissue was removed. The skin tissue was successively dehydrated with a gradient of ethanol from low to high concentrations and a gradient of *tert-*butanol, and the dehydrated skin tissue was adhered to a metal sample stage using conductive glue and dried in a critical point dryer. After drying, the sample was sputtered with ions at a current of 20 mA for 180 s, and finally, the tissue was gold-plated. The prepared sample was placed in the scanning electron microscope observation chamber, the sample environment was evacuated, and the observation field was moved to focus on the area to be measured. Collagen fibers were photographed at magnifications of 5000 times and 30 000 times.

### Transmission electron microscopy

Twenty-eight-day-old wound tissues were removed, and the tissue blocks were transferred to an electron microscope fixative solution, fixed and stored at 4°C for transportation. The tissue was refixed with 1% osmium tetroxide prepared in 0.1 M phosphate buffer (pH of 7.4) in the dark at room temperature for 2 h. The tissue was dehydrated successively with 30%–50%–70%–80%–95%–100%–100% alcohol for 20 min each time and with 100% acetone twice for 15 min each time. The ratio of acetone to the 812 embedding agent was 1:1 at 37°C for 2–4 hours and 1:2 at 37°C overnight for penetration. Pure 812 embedding agent was incubated at 37°C for 5–8 hours. The pure 812 embedding agent was poured into an embedding plate, and the sample was inserted into the embedding plate and then placed in a 37°C oven overnight. The embedding plate was placed in a 60°C oven for polymerization for 48 h, and the resin block was removed for standing. The resin block was sliced into ultrathin 60–80 nm slices by an ultrathin slicer, and the slices were picked up with a 150-mesh Fanghua film copper net. The copper net was stained with a 2% uranyl acetate saturated alcohol solution in the dark for 8 min, washed with 70% alcohol three times, washed with ultrapure water three times, stained with a 2.6% lead citrate solution without carbon dioxide for 8 min, and washed with ultrapure water three times. Then, the filter paper was slightly dried. The copper net slices were placed in a copper net box and dried overnight at room temperature. The slices were observed under a transmission electron microscope, and the images were analyzed.

### Atomic force microscopy

Frozen sections were prepared from 28-day-old wound tissues and subjected to mechanical analysis using a JPK NanoWizard 4 atomic force microscope. An MLCT probe was utilized, and the instrument was configured to operate in contact mode–force mapping mode. Parameters such as the indentation force, depth, and speed were optimized to determine the microscopic Young’s modulus. The mechanical data were analyzed using JPK SPM Data Processing software to derive the Young’s modulus values. For relaxation characteristics, the system was set to contact mode–spectroscopy force mode, with a protocol involving a 10-s hold period followed by retraction [49, 50]. The relaxation rate (RR) was calculated using the specified formula, and curves were generated using Origin software. Additionally, the contact mode–imaging mode was employed to acquire height and morphology maps, which were subsequently processed using JPK SPM Data Processing software to evaluate the microscopic skin morphology.

### Macroscopic mechanical properties

The skin was sampled from the mouse, and the detection area was 10 mm × 3 mm. The skin in the test area was stretched using MTS uniaxial material testing equipment. The skin in the test area was stretched to the specified length until it broke. The Young’s modulus curve of the skin was drawn using Origin software. The ratio of stress to strain was used to calculate the Young’s modulus to characterize the stiffness characteristics of the skin. The skin was stretched to 130% of its initial length and maintained. The change in the force value during the maintenance period was observed, and the relaxation rate was calculated as the ratio of stress to time to characterize the elasticity and resilience of the skin.

### Quantitative real-time RT–PCR

Total RNA was isolated from samples via TRIzol reagent (Invitrogen) following the manufacturer’s instructions. cDNAs were synthesized following DNase digestion (Takara). Reverse transcription was performed using oligo dT primers, and the resulting cDNA was diluted 1:100 for subsequent qPCR analysis. qPCR was conducted using SYBR Green Master Mix (Takara) in a 20 μL reaction volume. Relative gene expression was quantified using the 2^−ΔΔ^Ct method.

### Masson and sirius red staining

In accordance with the manufacturer’s instructions and the procedures of previous experiments, Masson’s trichrome blue (Solarbio, G1340) and sirius red (Solarbio, G1470) were used to stain the tissue slices.

### MDA and GSH/GSSG detection

In accordance with the manufacturer’s instructions, the MDA content in cells and tissues was detected using a lipid oxidation (MDA) detection kit (Beyotime, S0131S), and the ratio of GSH to GSSG in cells and tissues was detected using GSH and GSSG detection kits (Beyotime, S0053).

### Luciferase reporter assay

The cells were cotransfected with either wt-keap1–3’UTR or mut-keap1–3’UTR reporter plasmids along with the miR-29a-3p mimic or mimic NC. At 48 h post-transfection, cells were lysed, and the luciferase activity was measured using a Dual Luciferase Reporter Gene Assay Kit (Beyotime, RG027).

### Quantification and statistical analysis

Statistical analyses were performed using GraphPad Prism 7.0 software (GraphPad Software, San Diego, CA, USA). The normality of the data distribution was first assessed using the Shapiro–Wilk test. Normally distributed data were expressed as the mean ± SD and were analyzed by either unpaired Student’s t test (for comparisons between two groups) or one-way ANOVA (for multiple group comparisons), with Sidak’s multiple comparisons test applied as appropriate for post hoc analysis when ANOVA revealed significant differences. For the animal experiments, between-group differences were specifically evaluated using Sidak’s multiple comparisons test to compare the control group/Fb-exo group with the GW4869 group/T1DM group. *P* < .05 was considered statistically significant.

## Results

### Fibroblast exosomes promote wound healing by enhancing epidermal stem cell and fibroblast function

To investigate the role of fibroblast exosomes in cutaneous wound healing, we inhibited exosome production with GW4869 and administered exogenous fibroblast exosomes in a mouse excisional wound model. Compared with the control, GW4869 treatment significantly increased the unhealed wound area and reduced the neolpithelial tongue length on days 3, 7, and 10 post-wounding. Exogenous fibroblast exosomes reversed this delay in healing (Supplementary Figure S1a, b).

**Figure 1:**
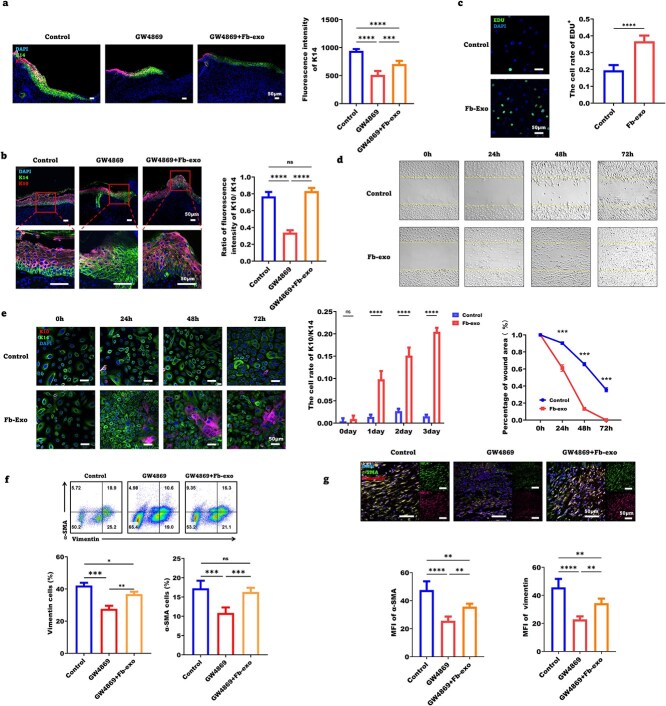
Fibroblast exosomes promote wound healing by enhancing epidermal stem cell and fibroblast function. (**a**) Representative images of the ratio of K10 to K14 fluorescence on Day 7 post-wounding in the control, GW4869, and GW4869 + Fb-exo groups. Statistical analysis of the K14 fluorescence intensity (scale bar = 50 μm; n = 5). (**b**) Representative images of the ratio of K10 terminal epidermal cells to K14 precursor cells in the control, GW4869, and GW4869 + Fb-exo groups. Statistical analysis of the ratio of K10 to K14 fluorescence intensity (scale bar = 50 μm; n = 5). (**c**) Determination of the proliferation of epidermal stem cells stimulated with fibroblast exosomes by EdU (scale bar = 50 μm; n = 5). (**d**) Evaluation of the motility of epidermal stem cells in the control and fibroblast exosome-stimulated groups by the scratch method (n = 3). (**e**) Immunofluorescence staining of K10 and K14 in epidermal stem cells stimulated with fibroblast exosomes for 0, 24, 48, and 72 h (scale bar = 50 μm; n = 3). (**f**) Representative fluorescence-activated cell sorting (FACS) images of vimentin and a-SMA + myofibroblasts in the control, GW4869-treated, and GW4869 + Fb-exo groups on the Day 7 post-wounding and statistical analysis (n = 3). (**g**) Immunofluorescence staining of vimentin and a-SMA in the skin of the control, GW4869-treated, and GW4869 + Fb-exo groups on Day 7 post-wounding and statistical analysis (scale bar = 50 μm; n = 3). Mean ± standard deviation (^*^P < 0.05; ^*^^*^P < 0.01; ^*^^*^^*^P < 0.001; ^*^^*^^*^^*^P < 0.0001; *ns* not statistically significant). *DAPI* 4′,6-Diamidino-2-phenylindole, *EDU* 5-ethynyl-2′-deoxyuridine, *K10* keratin 10, *K14* keratin 14, *MFI* mean fluorescence intensity, *α-SMA* α-smooth muscle actin

Wound re-epithelialization relies on the proliferation, migration, and differentiation of epidermal stem cells. On Day 7, the fluorescence intensity of K14, a marker of epidermal progenitors, was lower in the neoepithelium of GW4869-treated wounds than in that of control wounds but was restored by fibroblast exosomes (Figure 1a). The K10/K14 ratio, indicating epidermal differentiation, was reduced in GW4869 wounds on Day 10 but was normalized by exosomes (Figure 1b). To further elucidate the effects of fibroblast exosomes on epidermal stem cells, we performed in vitro studies with human epidermal stem cells. Fibroblast exosomes increased the percentage of EdU+ proliferating cells (Figure 1c), enhanced migration in scratch assays (Figure 1d), and promoted K10+ terminal differentiation (Figure 1e). These data suggest that fibroblast exosomes stimulate epidermal stem cell proliferation, migration, and differentiation to promote re-epithelialization.

Granulation tissue formation also contributes to wound healing. On Day 7, GW4869 reduced the granulation tissue collagen volume fraction and type I/III collagen ratio, which were restored by exosomes (Supplementary Figure S1c, d). FACS and immunofluorescence revealed that GW4869 decreased the percentage of vimentin+ fibroblasts and α-SMA+ myofibroblasts in granulation tissue on Day 7, whereas exosomes significantly increased these populations (Figure 1f, g). Together, these results demonstrate that fibroblast exosomes promote both re-epithelialization and granulation tissue formation to accelerate wound healing.

### Fibroblast exosomes significantly improve the epidermal barrier function of the healed skin

**Figure 2:**
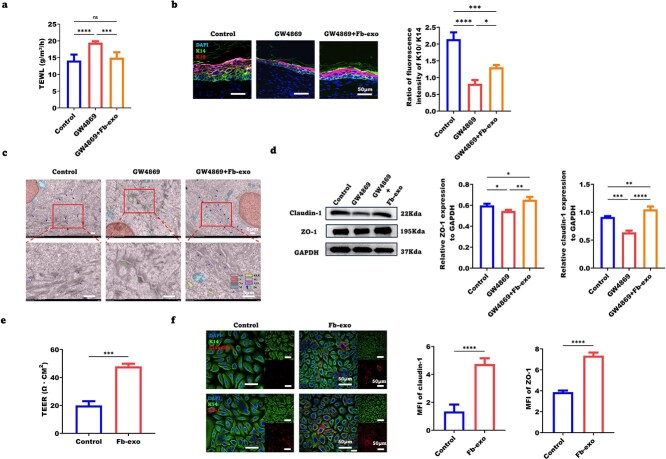
Fibroblast exosomes significantly improve the epidermal barrier function of healed skin. (**a**) Determination of the transepidermal water loss value of healed wounds in the control, GW4869-treated, and GW4869 + Fb-exo groups via statistical analysis (n = 6). (**b**) Representative images of the ratio of K10 terminal epidermal cells to K14 precursor cells after wound healing in the control, GW4869-treated, and GW4869 + Fb-exo groups (scale bar = 50 μm, n = 3). (**c**) Transmission electron microscopy observation of the microscopic structure of the epidermal layer of Day 28 wounds in the control, GW4869-treated, and GW4869 + Fb-exo groups (scale bar = 5 μm, n = 5). (**d**) Expression levels of the tight junction proteins claudin-1 and zo-1 in the healed wound tissue of the control, GW4869-treated, and GW4869 + Fb-exo groups, showing representative western blot (WB) images and statistical analysis (n = 3). (**e**) Changes in the transepithelial electrical resistance (TEER) of epidermal stem cells after stimulation with fibroblast exosomes for 24 h (n = 3). (**f**) Expression levels of tight junction proteins after stimulation with fibroblast exosomes for 24 h, as shown by representative IFC images and statistical analysis (scale bar = 50 μm, n = 5). Mean ± standard deviation (^*^P < 0.05; ^*^^*^P <0.01; ^*^^*^^*^P < 0.001; ^*^^*^^*^^*^P < 0.05; *ns* not statistically significant). *Cyt*  cytoplasm, *RER*  rough endoplasmic reticulum, *N*  nucleus, *SG*  secretion granules, nu  nucleolus, *ASS*  autophagy lysosome, *M*  mitochondria, *De*  desmosomes (normal = blue; abnormal = green), *TEWL* transepidermal water loss, *DAPI* 4′,6-Diamidino-2-phenylindole, *K10* keratin 10, *K14* keratin 14, *MFI* mean fluorescence intensity, *GAPDH* glyceraldehyde-3-phosphate dehydrogenase, *ZO-1* zonula Occludens-1, *TEER* transepithelial electrical resistance

We next assessed whether fibroblast exosomes affect the epidermal barrier of healed skin. On Day 28 post-wounding (14–21 days after complete healing), transepidermal water loss (TEWL) was greater in the healed skin of GW4869-treated mice than in that of control mice but was reduced by exosome treatment (Figure 2a). GW4869 decreased the epidermal thickness and K10/K14 ratio in healed skin, indicating impaired epidermal differentiation, but exosomes normalized these parameters (Supplementary Figure S2a, Figure 2b). Ultrastructurally, GW4869 caused widened intercellular spaces, reduced desmosomes, and sparse intracellular tension fibers in the healed epidermis, which were ameliorated by exosomes (Figure 2c).

The integrity of epidermal tight junctions is crucial for skin barrier function. Western blot analysis revealed that GW4869 decreased the expression of the tight junction proteins claudin-1 and ZO-1 in the healed epidermis, which was restored by the exosomes (Figure 2d). Immunohistochemistry confirmed these findings (Supplementary Figure S2b). To functionally assess barrier integrity, we measured transepithelial electrical resistance (TEER) in human epidermal stem cells. Fibroblast exosomes increased TEER (Figure 2e) and upregulated claudin-1 and ZO-1 expression (Figure 2f, Supplementary Figure S2c). Therefore, fibroblast exosomes improve epidermal differentiation and tight junctions to enhance skin barrier function.

### Fibroblast exosomes significantly improve the biomechanical properties of healed skin

**Figure 3:**
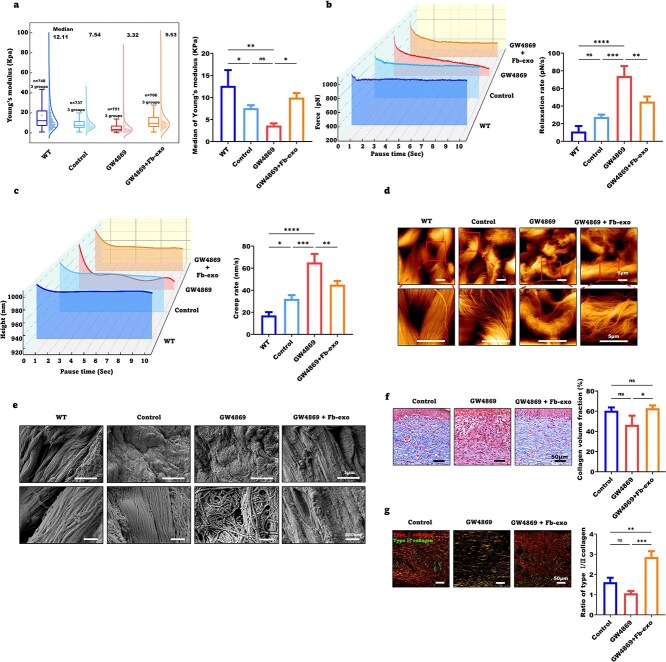
Fibroblast exosomes significantly improve the biomechanical properties of healed skin. (**a**) Atomic force microscopy was used to measure the distribution trend box plot of the Young’s modulus of collagen in the wounds on the Day 28 in the control, GW4869, and GW4869 + Fb-exo groups, and statistical analysis was performed (n = 3). (**b**) Atomic force microscopy was used to measure the stress relaxation rate curve of skin collagen, and statistical analysis was performed (n = 3). (**c**) Atomic force microscopy was used to measure the strain creep rate curve of skin collagen, and statistical analysis was performed (n = 3). (**d**) Atomic force microscopy was used to image the morphology of collagen fibers in frozen slices from the wounds of the control, GW4869, and GW4869 + Fb-exo groups on the 28th day (scale bar = 5 μm, n = 5). (**e**) Electron microscopy was used to image the morphology of collagen fibers in the wounds of the control, GW4869, and GW4869 + Fb-exo groups on Day 28 (scale bar = 5 μm, 500 nm; n = 5). (**f**) The collagen volume fraction of granulation tissue in the healed skin of the control, GW4869, and GW4869 + fb-exo groups was determined via Masson’s trichrome staining (scale bar = 50 μm, n = 3). (**g**) the proportions of type I and type III collagen in the healed skin of the control, GW4869, and GW4869 + Fb-exo groups were determined by sirius red staining (scale bar = 50 μm, n = 3). Mean ± standard deviation (^*^P < 0.05; ^*^^*^P < .0.01; ^*^^*^^*^P < 0.001; ^*^^*^^*^^*^P < 0.0001; *ns* not statistically significant). *WT* wild type

Cutaneous scarring impacts the biomechanical properties of healed skin. Atomic force microscopy (AFM) revealed that Young’s modulus, a measure of stiffness, was lower on Day 28 in the healed skin of GW4869-treated mice than in that of the control mice. Conversely, the relaxation rate and strain creep, which are measures of viscoelastic properties, were greater in the skin of GW4869-treated mice. Fibroblast exosomes significantly improved these abnormal biomechanical parameters (Figure 3a–c).

The biomechanical properties of skin are determined mainly by the dermal extracellular matrix, particularly collagen fibers. AFM and scanning electron microscopy (SEM) revealed that collagen fiber bundling and alignment in the healed dermis progressively decreased from the uninjured skin to the control, exosome-treated, and GW4869-treated groups (Figure 3d, e). Masson’s trichrome and picrosirius red staining demonstrated that GW4869 reduced the collagen volume fraction and type I/III collagen ratio, respectively, in the healed dermis, whereas exosomes increased these parameters (Figure 3f, g). Immunohistochemistry confirmed that GW4869 decreased dermal type I and III collagen levels, which were increased by exosomes (Supplementary Figure S3a). These findings indicate that fibroblast exosomes enhance collagen deposition and remodeling to improve the biomechanical strength of healed skin.

Biomechanical evaluation on Day 40 post-wounding revealed that GW4869-treated skin presented a significantly reduced Young’s modulus and decreased slack rate, indicating impaired stiffness and elastic recovery capability. In contrast, the fibroblast exosome treatment group effectively counteracted the effects of GW4869; the Young’s modulus of the fibroblast exosome treatment group was significantly greater than that of the GW4869 group, with a slack rate approaching that observed in normal wound healing (Supplementary Figure S3b, c). These findings demonstrate that fibroblast exosomes play a crucial role in improving the mechanical properties of healed wounds.

### Fibroblast exosomes promote re-epithelialization of diabetic wounds and improve ECM deposition

**Figure 4:**
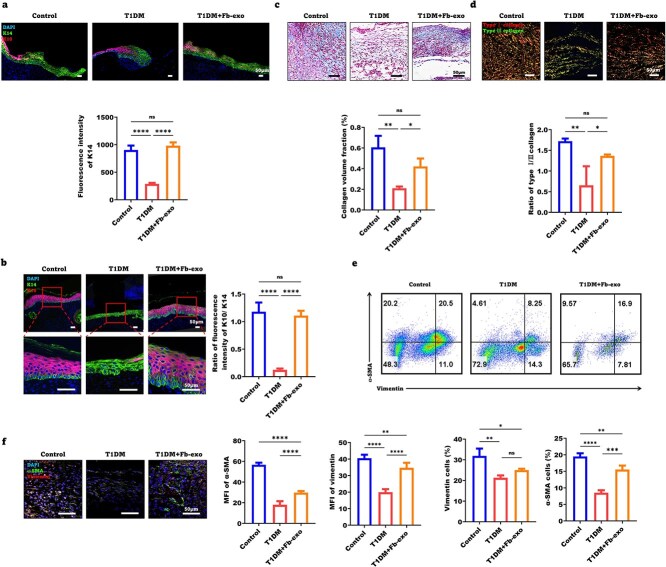
Fibroblast exosomes promote re-epithelialization of diabetic wounds and improve ECM deposition. (**a**) Representative images of the ratio of K10 to K14 fluorescence on Day 7 after wounding in the control, T1DM and T1DM + Fb-exo groups. Statistical analysis of the K14 fluorescence intensity (scale bar = 50 μm, n = 5). (**b**) Representative images of the ratio of K10 terminal epidermal cells to K14 progenitor cells in the control, T1DM and T1DM + Fb-exo groups. Statistical analysis of the ratio of K10 and K14 fluorescence intensities (scale bar = 50 μm, n = 5). (**c**) Masson’s trichrome staining was used to determine the collagen volume fraction in the granulation tissue of the wounds in the control, T1DM and T1DM + Fb-exo groups (scale bar = 50 μm, n = 3). (**d**) Sirius red staining was used to determine the ratio of type I to type III collagen in the granulation tissue of the wounds in the control, T1DM and T1DM + Fb-exo groups (scale bar = 50 μm, n = 3). (**e**) Representative FACS images of vimentin and α-SMA+ myofibroblasts on Day 7 after wound creation in the control, T1DM and T1DM + Fb-exo groups, and statistical analysis was performed (n = 3). (**f**) Immunofluorescence staining of vimentin and α-SMA on Day 7 after skin wound creation in the control, T1DM and T1DM + Fb-exo groups is shown, and statistical analysis was performed (scale bar = 50 μm, n = 5). Mean ± standard deviation (^*^P < 0.05; ^*^^*^P <0.01; ^*^^*^^*^P < 0.001; ^*^^*^^*^^*^P < 0.0001; *ns* not statistically significant). *DAPI* 4′,6-Diamidino-2-phenylindole, *K10* keratin 10, *K14* keratin 14, *MFI* mean fluorescence intensity, *α-SMA* α-smooth muscle actin

Diabetes significantly impairs wound healing. Compared with nondiabetic control mice, both type 1 diabetic mellitus (T1DM) and type 2 diabetic mellitus (T2DM) mice presented markedly delayed wound closure (Supplementary Figure S4a), which was significantly improved by fibroblast-derived exosome treatment. In terms of epidermal stem cell function, both T1DM and T2DM led to reduced K14 fluorescence intensity on Day 7 post-wounding and a decreased K10/K14 ratio on Day 10, suggesting impaired epidermal stem cell function. Notably, exosome treatment effectively reversed these functional deficits (Figure 4a, b, Supplementary Figure S4c, d). With respect to fibroblast activation, fibroblast activity was compromised in both T1DM and T2DM wounds on Day 7, and exosome treatment significantly alleviated this impairment (Figure 4f, Supplementary Figure S4e). Given that T1DM and T2DM wounds exhibited similar cellular dysfunction during healing, we further focused on T1DM for subsequent investigations. Notably, we observed that T2DM wounds presented lower oxidative stress levels than did T1DM wounds, which also contributed to our selection of T1DM for further mechanistic studies (Supplementary Figure S6a–c).

T1DM wounds presented a decreased collagen volume fraction and type I/III collagen ratio in Day 7 granulation tissue, which were restored by exosomes (Figure 4c, d). The percentages of vimentin+ fibroblasts and α-SMA+ myofibroblasts were lower in T1DM mouse wounds but increased after exosome treatment, as shown by FACS (Figure 4e) and immunofluorescence (Figure 4f). Therefore, fibroblast exosomes rescue impaired re-epithelialization and ECM deposition in diabetic wounds.

### Fibroblast exosomes significantly improve the epidermal barrier function of healed skin in diabetic wounds

**Figure 5:**
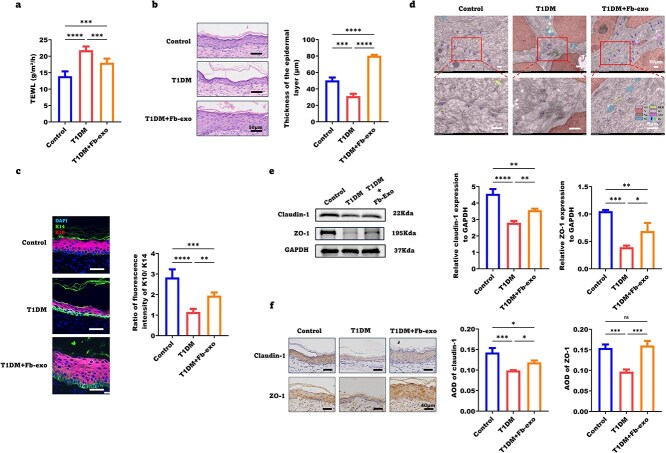
Fibroblast exosomes significantly improve the epidermal barrier function of healed skin in diabetic wounds. (**a**) Measurement and statistical analysis of the transepidermal water loss values of the healed wounds of the mice in the control, T1DM and T1DM + Fb-exo groups (n = 6). (**b**) Representative images of the wound epidermal thickness of the mice in the control, T1DM and T1DM + Fb-exo groups on Day 28 and statistical analysis (scale bar = 50 μm, n = 3). (**c**) Representative images of the ratio of K10 terminal epidermal cells to K14 progenitor cells in the control, T1DM and T1DM + Fb-exo groups after wound healing (scale bar = 50 μm, n = 5). (**d**) Representative transmission electron microscopy images of the wound epidermal microstructure of the mice in the control, T1DM and T1DM + Fb-exo groups on Day 28 (scale bar = 5 μm, n = 5). (**e**, **f**) the expression levels of the tight junction proteins claudin-1 and zo-1 in the wound tissues of the mice in the control, T1DM and T1DM + Fb-exo groups after healing. Representative WB and IHC images are shown, and statistical analysis was performed (scale bar = 50 μm, n = 3). Mean ± standard deviation (^*^P < 0.05; ^*^^*^P < 0.01; ^*^^*^^*^P <0.001; ^*^^*^^*^^*^P < 0.0001; *ns* not statistically significant). *Cyt*  cytoplasm, *RER*  rough endoplasmic reticulum, *N*  nucleus, *SG*  secretion granules, *nu*  nucleolus, *ASS*  autophagy lysosome, *M*  mitochondria, *De*  desmosomes (normal = blue; abnormal = green). *T1DM* type 1 diabetes mellitus, *TEWL* transepidermal water loss, *DAPI* 4′,6-Diamidino-2-phenylindole, *K10* keratin 10, *K14* keratin 14, *GAPDH* glyceraldehyde-3-phosphate dehydrogenase, *ZO-1* zonula Occludens-1, *AOD* average optical density

We further examined whether fibroblast exosomes improve the epidermal barrier of diabetic wounds. On Day 28 post-injury, TEWL was elevated in the healed skin of T1DM mice compared with that of control mice but was reduced by exosome treatment (Figure 5a). The thickness and K10/K14 ratio of the healed neoepidermis were reduced in T1DM mice, indicating impaired differentiation, but these parameters were significantly increased by exosomes (Figure 5b, c).

Ultrastructurally, T1DM caused widened intercellular spaces, fewer desmosomes, and sparse tension fibers in the healed epidermis, which were ameliorated by exosomes (Figure 5d). Western blot analysis revealed that exosomes upregulated claudin-1 and ZO-1 in the T1DM healed epidermis to levels approaching those in the nondiabetic control epidermis (Figure 5e). Immunohistochemistry confirmed these findings (Figure 5f). These data show that fibroblast exosomes enhance epidermal differentiation and tight junctions to restore skin barrier integrity in diabetic wounds.

### Fibroblast exosomes significantly improve the biomechanical properties of healed skin in diabetic wounds

**Figure 6:**
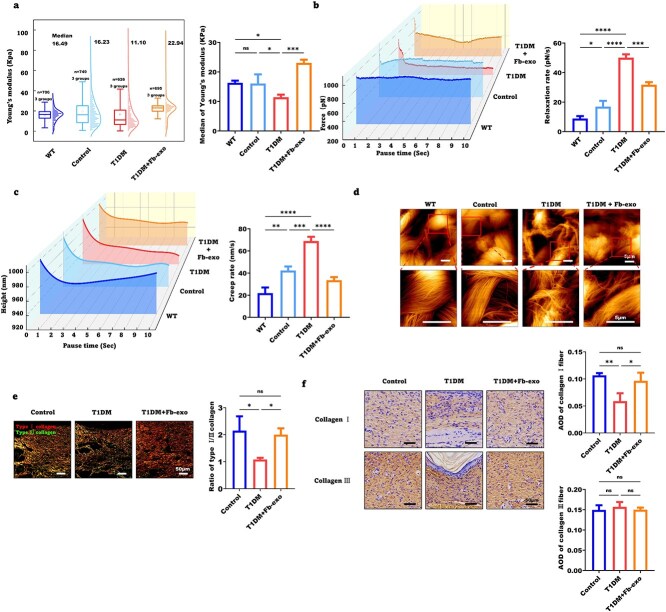
Fibroblast exosomes significantly improve the biomechanical properties of healed skin in diabetic wounds. (**a**) Box plot and statistical analysis of the distribution trend of collagen Young’s modulus bands in wounds of mice in the control, T1DM and T1DM + Fb-exo groups on Day 28, as measured by atomic force microscopy (n = 3). (**b**) Skin collagen stress relaxation rate curve measured by atomic force microscopy and statistical analysis (n = 3). (**c**) Skin collagen strain creep rate curve measured by atomic force microscopy and statistical analysis (n = 3). (**d**) Atomic force microscopy images of collagen fiber morphology obtained from frozen sections of wounds from mice in the control, T1DM and T1DM + Fb-exo groups on Day 28 (scale bar = 5 μm, n = 5). (**e**) Sirius red staining was used to determine the ratio of type I to type III collagen in the granulation tissue of the healed wounds of the mice in the control, T1DM and T1DM + Fb-exo groups (scale bar = 50 μm, n = 3). (**f**) The expression levels of type I and type III collagen in the granulation tissue of the healed wounds of the mice in the control, T1DM and T1DM + Fb-exo groups were determined by IHC (scale bar = 50 μm, n = 3). Mean ± standard deviation (^*^P <0.05; ^*^^*^P < 0.01; ^*^^*^^*^P < 0.001; ^*^^*^^*^^*^P < 0.0001; *ns* not statistically significant). *T1DM* type 1 diabetes mellitus, *WT* wild type, *AOD* average optical density

Diabetes impairs biomechanical wound healing. AFM revealed a reduced Young’s modulus and elevated stress relaxation and strain creep rates in the healed skin of T1DM mice on Day 28, indicating decreased stiffness and elasticity. These abnormalities were improved by the addition of fibroblast exosomes (Figure 6a–c).

AFM (Figure 6d) and SEM (Supplementary Figure S5a) shows that healed skin collagen fiber bundling and alignment progressively decreased from the nondiabetic group to the exosome-treated and untreated diabetic groups. The collagen volume fraction and type I/III collagen ratio were lower in the T1DM-healed dermis than in the control dermis according to Masson’s trichrome (Supplementary Figure S5b) and picrosirius red (Figure 6e) staining, respectively, and these ratios were increased by exosomes. Immunohistochemistry revealed that the mean optical density of type I collagen was decreased in T1DM mice and rescued by exosomes, whereas type III collagen was unchanged across groups (Figure 6f). Therefore, fibroblast exosomes stimulate collagen deposition and remodeling to increase diabetic wound healing.

Longitudinal biomechanical analysis at 40 days post-wounding revealed that T1DM wounds exhibited persistent impairments in both tissue stiffness and viscoelastic properties compared with normoglycemic controls. Importantly, fibroblast exosome therapy significantly attenuated these diabetes-associated biomechanical deficits, restoring wound mechanical competence to near-physiological levels (Supplementary Figure S5c, d).

### Fibroblast exosomes improve wound healing quality by reducing oxidative stress damage

**Figure 7:**
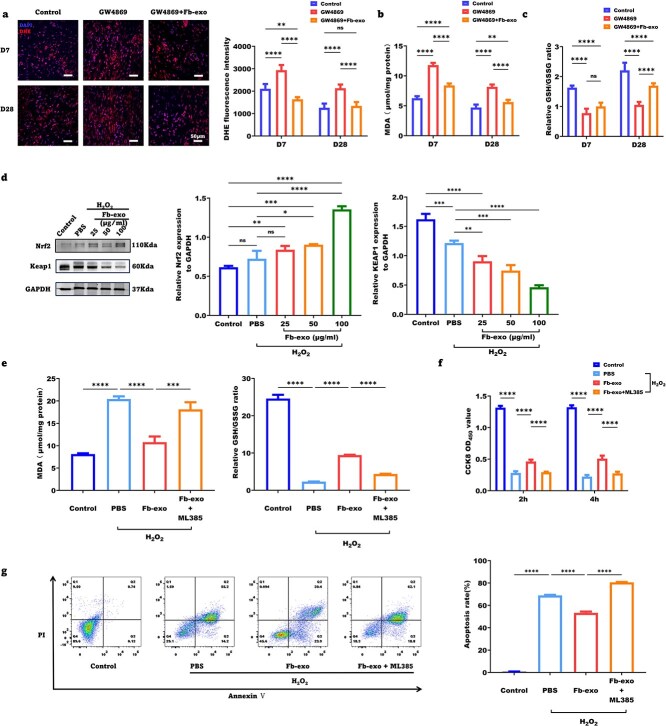
Fibroblast exosomes improve wound healing quality by reducing oxidative stress damage. (**a**) Representative images of DHE staining and statistical analysis of the fluorescence intensity of fresh frozen tissue sections from wounds in the control, GW4869 and GW4869 + Fb-exo groups on Days 7 and 28 post-wounding (scale bar = 50 μm, n = 5). (**b**) Statistical analysis of the MDA concentration in wound tissue from the control, GW4869 and GW4869 + Fb-exo groups on Days 7 and 28 post-wounding (n = 5). (**c**) Statistical analysis of the ratio of GSH/GSSG in wound tissue in the control, GW4869 and GW4869 + Fb-exo group on Days 7 and 28 post-wounding (n = 5). (**d**) Representative images of protein immunoblot bands of KEAP1 and Nrf2 in epidermal stem cells and statistical analysis (n = 3). (**e**) Statistical analysis of the MDA concentration and the GSH/GSSG ratio after epidermal stem cells were stimulated with fibroblast exosomes under oxidative stress conditions (n = 3). (**f**) Statistical analysis of cell viability after epidermal stem cells were stimulated with fibroblast exosomes under oxidative stress conditions (n = 3). (**g**) Representative flow cytometry images and statistical analysis of cell apoptosis detected by Annexin V/PI double staining of epidermal stem cells after stimulation with fibroblast exosomes under oxidative stress conditions (n = 3). Mean ± standard deviation (^*^P <0.05; ^*^^*^P < 0.01; ^*^^*^^*^P <0.001; ^*^^*^^*^^*^P < 0.0001; *ns* not statistically significant). *DAPI* 4′,6-Diamidino-2-phenylindole, *DHE* dihydroethidium, *MDA* malondialdehyde, *GSH* glutathione, *GSSG* glutathione Disulfide, *Nrf2* nuclear factor erythroid 2-related factor 2, *KEAP1* Kelch-like ECH-associated protein 1, *GAPDH* glyceraldehyde-3-phosphate dehydrogenase, *CCK8* cell counting kit-8, *PI* propidium iodide

Oxidative stress impairs wound healing. The DHE fluorescence intensity, an indicator of ROS, was greater for Day 7 wounds than for Day 28 healed skin from GW4869-treated (Figure 7a) and T1DM mice (Supplementary Figure S6a). Fibroblast exosomes significantly reduced ROS in both models. The levels of malondialdehyde (MDA), a marker of lipid peroxidation, were increased by GW4869 and diabetes but restored by exosomes (Figure 7b, Supplementary Figure S6b).

Glutathione is a major cellular antioxidant. The ratio of reduced to oxidized glutathione (GSH/GSSG), which reflects the antioxidant capacity, was decreased in wounds and healed skin by GW4869 and diabetes but was restored by exosomes (Figure 7c, Supplementary Figure S6c).

The Kelch-like ECH-associated protein 1 (KEAP1)/nuclear factor erythroid 2-related factor 2 (Nrf2) pathway is a master regulator of cellular antioxidant responses. Western blot analysis revealed that H2O2 increased KEAP1 expression and decreased Nrf2 expression in cultured human epidermal stem cells, and these effects were reversed by fibroblast exosomes in a concentration-dependent manner (Figure 7d). Nrf2 inhibition with ML385 abolished the antioxidant effects of the exosomes, increasing the MDA content and reducing the GSH/GSSG ratio (Figure 7e).

H_2_O_2_ impaired epidermal stem cell proliferation and induced apoptosis, which were ameliorated by exosomes in an Nrf2-dependent manner. A CCK-8 assay revealed that H_2_O_2_ inhibited cell proliferation at 2 h and 4 h, whereas exosomes promoted proliferation. ML385 pretreatment weakened the proproliferative effect of the exosomes (Figure 7f). Annexin V/PI flow cytometry revealed that H_2_O_2_ increased cell apoptosis, which was reduced by exosomes. ML385 abolished the antiapoptotic effect of the exosomes (Figure 7g). Collectively, these results suggest that fibroblast exosomes activate KEAP1/Nrf2 signaling in epidermal stem cells to neutralize oxidative stress and maintain cell function, thereby promoting wound healing.

### Fibroblast exosomes increase the capacity for cellular antioxidant stress by promoting the degradation of *KEAP1* mRNA via miR-29a-3p

**Figure 8:**
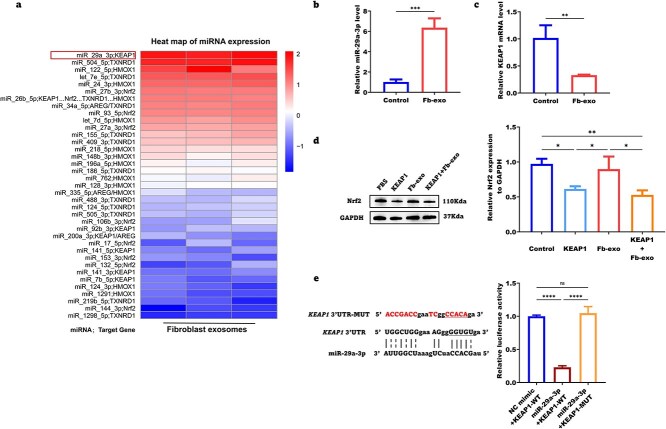
Fibroblast exosomes increase the capacity for cellular antioxidant stress by suppressing *KEAP1* gene transcription via miR-29a-3p. (**a**) Enrichment heatmap of miRs associated with oxidative stress pathways in fibroblast-derived exosomes. (**b**) Intracellular miR-29a-3p levels in epidermal stem cells following the phagocytosis of fibroblast-derived exosomes (n = 3). (**c**) Intracellular *KEAP1* mRNA expression levels in epidermal stem cells following phagocytosis of fibroblast-derived exosomes (n = 3). (**d**) Representative western blot analysis images of Nrf2 degradation in response to recombinant KEAP1 protein and fibroblast exosomes (n = 3). (**e**) Prediction of miR-29a-3p binding sites and measurement of relative luciferase activity in epidermal stem cells cotransfected with the miR-29a-3p mimic or negative control mimic and WT or Mut Keap1 vectors (n = 3). (^*^P < 0.05; ^*^^*^P < 0.01; ^*^^*^^*^P < 0.001; ^*^^*^^*^^*^P <0.0001; *ns* not statistically significant). *Nrf2* nuclear factor erythroid 2-related factor 2, *KEAP1* Kelch-like ECH-associated protein 1, *GAPDH* glyceraldehyde-3-phosphate dehydrogenase, *MUT* mutant

Through high-throughput sequencing analysis of fibroblast-derived exosomes, we identified miR-29a-3p as a key miRNA associated with oxidative stress (Figure 8a). Subsequent qPCR validation revealed a significant increase in intracellular miR-29a-3p levels in epidermal stem cells following treatment with fibroblast exosomes (Figure 8b). These results suggest that fibroblast-derived exosomes may regulate cellular oxidative stress responses by delivering miR-29a-3p to recipient cells.

To further explore the targets of miR-29a-3p, we analyzed the expression levels of KEAP1 mRNA in cells treated with fibroblast exosomes using qPCR. The results revealed that fibroblast exosomes significantly reduced the intracellular expression of KEAP1 mRNA, suggesting that fibroblast-derived exosomes may inhibit KEAP1 gene transcription or mRNA stability through miR-29a-3p (Figure 8c). Western blot results demonstrated that recombinant KEAP1 protein significantly promoted Nrf2 degradation, which was consistent with its function in mediating Nrf2 degradation through the ubiquitin–proteasome pathway. However, exogenous supplementation with fibroblast-derived exosomes was insufficient to counteract the degradation of Nrf2 induced by recombinant KEAP1 protein (Figure 8d). Using RNAhybrid, we predicted potential binding sites between miR-29a-3p and Keap1. A luciferase reporter assay demonstrated that the miR-29a-3p mimic significantly suppressed firefly luciferase expression. However, this inhibitory effect was abolished when the Keap1 mutant was used (Figure 8e). These findings suggest that fibroblast exosomes may primarily regulate KEAP1 through miR-29a-3p, which targets the 3’UTR of its mRNA to promote degradation, with limited effects on the degradation of the KEAP1 protein. Consistent results were also obtained through CCK-8 assays (Figure S6d).

## Discussion

### Comprehensive analysis of the effects of fibroblast exosomes on wound healing

Our study provides a comprehensive analysis of the effects of fibroblast exosomes on both the epidermal and dermal components of wound healing. Unlike previous studies that focused on specific aspects, such as angiogenesis [51] or fibroblast [52] function, our research demonstrates the multifaceted roles of fibroblast exosomes in promoting re-epithelialization, enhancing extracellular matrix (ECM) deposition, and improving the quality of healed skin. This comprehensive approach allows for a more holistic understanding of how fibroblast exosomes orchestrate the complex process of wound healing.

Our findings are consistent with previous reports showing that exosomes derived from various cell types, including mesenchymal stem cells (MSCs) [53], adipose-derived stem cells (ASCs) [54], and keratinocytes [10], can promote wound healing. However, these studies often focused on specific aspects of wound healing, such as angiogenesis [55], collagen deposition [56], or immunomodulation [57]. In contrast, our study provides a more comprehensive assessment of the effects of fibroblast exosomes on both epidermal and dermal wound healing processes.

For example, we demonstrated that fibroblast exosomes promote re-epithelialization by stimulating epidermal stem cell proliferation, migration, and differentiation. These findings agree with those of previous studies, which showed that exosomes can enhance keratinocyte proliferation and migration [10,58]. However, our study specifically investigated the effects of fibroblast exosomes on epidermal stem cells, which are the primary drivers of re-epithelialization [24].

In addition to their effects on re-epithelialization, we showed that fibroblast exosomes promote granulation tissue formation by increasing fibroblast abundance and myofibroblast activation. These findings are consistent with previous reports demonstrating the importance of fibroblasts and myofibroblasts in ECM deposition and wound contraction [59,60]. However, our study is unique in demonstrating the direct effects of fibroblast exosomes on these key cellular components of granulation tissue.

Furthermore, our comprehensive assessment of healed skin quality, including epidermal barrier function, biomechanical properties, and resistance to oxidative stress, sets our study apart from previous work. While some studies have investigated the effects of exosomes on individual aspects of skin quality, such as the collagen content [26] or hydration [61], our study provides a more holistic evaluation of healed skin integrity.

Despite these strengths, our study has several limitations that warrant further investigation. First, we used a mouse model of excisional wounds, which may not fully recapitulate the complexity of chronic human wounds. Future studies should validate our findings in more clinically relevant models, such as diabetic foot ulcers or pressure ulcers. Second, while we demonstrated the effects of fibroblast exosomes on key cell types involved in wound healing (i.e. epidermal stem cells and fibroblasts), we did not investigate their potential impact on other important players, such as immune cells and endothelial cells. Future research should explore the broader cellular targets and mechanisms of action of fibroblast exosomes in wound healing.

### Novel insights into epidermal stem cell regulation by fibroblast exosomes

Our study provides novel insights into how fibroblast exosomes directly modulate epidermal stem cell function. We demonstrated that fibroblast exosomes increase the proliferation, migration, and differentiation of epidermal stem cells, which are the primary drivers of re-epithelialization. These findings provide a mechanistic explanation for the enhanced re-epithelialization observed with exosome treatment and represent a unique contribution to the field.

Previous studies have shown that exosomes derived from various cell types, such as MSCs [62] and keratinocytes [10], can promote keratinocyte proliferation and migration. However, these studies often used heterogeneous populations of keratinocytes and did not specifically investigate the effects of exosomes on epidermal stem cells. In contrast, our study focused on the direct impact of fibroblast exosomes on epidermal stem cell function, which is a novel aspect of our research.

Our findings are consistent with those of recent studies demonstrating the importance of epidermal stem cells in wound re-epithelialization. For example, Aragona et al. reported that epidermal stem cells are essential for wound closure and that their depletion leads to impaired re-epithelialization [25]. Similarly, Joost et al. demonstrated that epidermal stem cells are the primary contributors to wound re-epithelialization and that their proliferation and differentiation are critical for this process [63]. Our study extends these findings by showing that fibroblast exosomes can directly modulate epidermal stem cell function to promote re-epithelialization.

The mechanisms by which fibroblast exosomes regulate epidermal stem cell function remain to be fully elucidated. However, our findings suggest that the activation of the KEAP1/Nrf2 antioxidant pathway may play a role. We demonstrated that fibroblast exosomes increase Nrf2 expression and reduce oxidative stress in epidermal stem cells, which is consistent with previous reports showing that Nrf2 activation promotes keratinocyte proliferation and migration [64]. Future studies should investigate the specific cargo (e.g. microRNAs and proteins) within fibroblast exosomes that mediate their effects on epidermal stem cells and explore additional signaling pathways that may be involved.

One limitation of our study is that we focused on the effects of fibroblast exosomes on epidermal stem cells in the context of wound healing. However, epidermal stem cells also play critical roles in skin homeostasis and regeneration under physiological conditions [22]. Future research should investigate whether fibroblast exosomes modulate epidermal stem cell function in non-wounded skin and explore the potential implications for skin aging and regeneration.

### Comprehensive assessment of healed skin quality

A significant strength of our study is the comprehensive assessment of healed skin quality, which goes beyond simply measuring wound closure rates. Current research on exosomes predominantly relies on evidence from acute wound models, with insufficient investigations into their long-term effects on chronic wound healing. Moreover, studies have largely focused on mesenchymal stem cell-derived exosomes, whereas the prolonged efficacy of exosomes from other cellular sources remains systematically unexplored. We evaluated multiple parameters of healed skin integrity, including epidermal barrier function, biomechanical properties, and resistance to oxidative stress. Our findings demonstrate that fibroblast exosome treatment not only accelerates wound closure but also improves the long-term quality of healed skin. This comprehensive assessment of healed skin quality is a unique aspect of our research that sets it apart from previous studies.

Our results showing improved epidermal barrier function with fibroblast exosome treatment are consistent with recent studies demonstrating the importance of exosomes in regulating skin barrier integrity. For example, Choi et al. reported that ASC-derived exosomes improve skin hydration and barrier function in a mouse model of atopic dermatitis [61]. Similarly, Kim et al. demonstrated that MSC-derived exosomes enhance skin barrier function by increasing the expression of tight junction proteins, such as claudin-1 and ZO-1 [65]. Our study extends these findings by showing that fibroblast exosomes specifically improve epidermal barrier function in the context of wound healing.

In addition to barrier function, we assessed the biomechanical properties of healed skin, which are largely determined by the composition and organization of the ECM. Our findings showing increased collagen deposition and improved biomechanical strength with fibroblast exosome treatment are consistent with previous reports demonstrating the importance of collagen in skin mechanics [66]. However, our study is unique in demonstrating the direct effects of fibroblast exosomes on collagen deposition and remodeling in healed skin.

Furthermore, our assessment of oxidative stress in healed skin is novel. Oxidative stress is known to impair wound healing and contribute to the formation of chronic wounds [67]. Our findings of reduced oxidative damage and increased antioxidant capacity with fibroblast exosome treatment suggest that exosomes may promote wound healing, in part, by mitigating oxidative stress. This finding is consistent with previous studies demonstrating the antioxidant effects of exosomes derived from various cell types [68,69].

In our study, we evaluated the long-term biomechanical properties of healed wounds through Young’s modulus and stress relaxation measurements, which demonstrated the therapeutic efficacy of fibroblast-derived exosomes. These findings align with current research investigating the potential regulatory role of exosomes in chronic wound repair. In a recent study, Wang et al. developed an adipose-derived mesenchymal stem cell exosome–hydrogel composite system that not only promoted the orderly arrangement of collagen fibers at the wound site but also demonstrated a time-dependent increase in collagen deposition [52]. This dynamically balanced ECM remodeling process may provide long-term benefits for improving the mechanical strength of healed tissue. Furthermore, in a 12-week clinical trial, the sustained efficacy of adipose-derived stem cell exosomes, including increased collagen synthesis and improved skin barrier function, was consistent with these findings, suggesting that adipose-derived stem cell exosomes may influence long-term tissue remodeling by maintaining ECM homeostasis [70]. In our experiments, fibroblast exosomes were observed to enhance the mechanical strength, deformation resistance, and external force buffering capacity of healed skin in mice. Notably, miR-29a-3p, an effector molecule within fibroblast exosomes, has been shown to play a significant role in regulating fibrosis in the heart, liver, and lungs, providing further theoretical support for the long-term prognostic benefits of fibroblast exosomes in wound healing [71–73]. Currently, our experiments are limited to murine wound models. Given the differences between murine and human wound healing, future work will focus on extending the observation period to large animal models, human skin models, or clinical samples to evaluate long-term outcomes such as scar formation, skin elasticity, and collagen remodeling, while also incorporating other critical skin quality indicators, such as nerve regeneration and pigmentation. Additionally, multicenter clinical studies should be conducted to further validate the long-term safety and efficacy of exosome therapy, paving the way for broader clinical applications.

### Mechanistic insights into the antioxidant effects of fibroblast exosomes

Our study provides novel insights into the molecular mechanisms underlying the antioxidant effects of fibroblast exosomes. We demonstrated that fibroblast exosomes reduce oxidative stress in epidermal stem cells by activating the KEAP1/Nrf2 antioxidant pathway. Specifically, we showed that exosome treatment increases Nrf2 expression and decreases KEAP1 levels in a concentration-dependent manner, leading to enhanced antioxidant responses and reduced oxidative damage. These findings demonstrate the protective effects of fibroblast exosomes against oxidative stress and reveal a specific molecular pathway through which exosomes exert their antioxidant effects. This mechanistic insight is a unique contribution to our study that expands our understanding of how exosomes promote wound healing.

Our results are consistent with those of previous studies demonstrating the antioxidant effects of exosomes derived from various cell types. For example, Kubota et al. reported that exosomes derived from endogenous bone marrow-derived MSCs reduce oxidative stress in a diabetic rat model [74]. Similarly, Niu et al. demonstrated that exosomes derived from bone marrow MSCs attenuate oxidative stress and improve skin flap survival in a rat model [75]. However, these studies did not investigate the specific molecular pathways underlying the antioxidant effects of exosomes.

Our findings on the involvement of the KEAP1/Nrf2 pathway in mediating the antioxidant effects of fibroblast exosomes are consistent with previous reports demonstrating the importance of this pathway in wound healing. For example, Li et al. reported that activation of the Nrf2 pathway promotes wound healing by reducing oxidative stress and inflammation in a rat model of diabetic wounds [76]. Similarly, Wu et al. demonstrated that Nrf2 activation promotes keratinocyte proliferation and migration, which are critical for re-epithelialization [77]. Our study extends these findings by showing that fibroblast exosomes can activate the KEAP1/Nrf2 pathway to exert their antioxidant and pro-healing effects.

Previous studies have demonstrated that exosomes can alleviate cellular oxidative stress through specific cargo by activating the Nrf2 signaling pathway. For example, Wang et al. reported that MSC-derived exosomal miR-135a activates the Nrf2 pathway to reduce oxidative stress and promote wound healing in a diabetic mouse model [18]. Our experimental data demonstrated that fibroblast-derived exosomes contain miR-29a-3p, which plays a critical role in suppressing KEAP1 gene transcription. This suppression leads Nrf2 pathway activation, thereby enhancing cellular antioxidant responses. This finding is consistent with previous research conclusions. For example, Zhou et al. reported that high glucose levels in diabetic rats led to a downregulation of miR-29a-3p expression, which subsequently increased Keap1 expression and ultimately induced renal tubular epithelial injury [78]. Our findings not only confirmed the importance of miRNAs in exosome-mediated antioxidant effects but also identified miR-29a-3p as a key regulator of the KEAP1/Nrf2 pathway in fibroblast-derived exosomes. Future studies should further investigate the therapeutic potential of miR-29a-3p and other cargo within fibroblast exosomes, as well as their broader implications in oxidative stress-related diseases.

This study demonstrated that miR-29a-3p exerts its antioxidant effects primarily by modulating the core antioxidant pathway, the KEAP1/Nrf2 axis. However, emerging evidence suggests that miR-29a-3p may regulate a broader antioxidant network. For example, miR-29a-3p may influence the expression of antioxidant enzymes by modulating DNA methyltransferases (DNMTs). As demonstrated in a study by Zheng et al., miR-29a-3p can increase mitochondrial superoxide dismutase (SOD2) expression through epigenetic regulation of DNMT3A, thereby reducing intracellular ROS levels, rescuing mitochondrial dysfunction, and preventing cellular senescence [73]. Additionally, miR-29a-3p may upregulate glutathione metabolism-related genes such as GPx4 and interact with inflammatory pathways such as NF-κB, further lowering ROS levels and enhancing the cellular antioxidant capacity [79,80]. These findings suggest that miR-29a-3p may coordinate antioxidant responses through multiple targets or molecular axes, warranting further investigation. Nevertheless, our current data strongly support KEAP1/Nrf2 as the dominant pathway mediating the antioxidant effects of miR-29a-3p in fibroblast exosomes on epidermal stem cells.

One limitation of our study is that we focused on the effects of fibroblast exosomes on oxidative stress in epidermal stem cells. However, oxidative stress also affects other cell types involved in wound healing, such as fibroblasts and endothelial cells [81]. Future research should investigate the potential antioxidant effects of fibroblast exosomes on these other cell types and explore their contributions to wound healing. Additionally, although we demonstrated that fibroblast-derived exosomes enhance cellular antioxidant capacity by regulating the KEAP1-Nrf2 pathway through the degradation of KEAP1 mRNA through miR-29a-3p, whether miR-29a-3p concurrently modulates other antioxidant molecules, such as PI3K/AKT or MAPK, warrants further investigation.

### Therapeutic potential in diabetic wound healing

Our study highlights the therapeutic potential of fibroblast exosomes for diabetic wound healing. We demonstrated that fibroblast exosome treatment accelerates wound closure, promotes re-epithelialization, enhances ECM deposition, and improves the quality of healed skin in a mouse model of type 1 diabetes. These findings suggest that the use of fibroblast exosomes may be a promising therapeutic strategy for the treatment of diabetic wounds, which are a major clinical challenge associated with significant morbidity and mortality [33].

Our results are consistent with those of previous studies demonstrating the efficacy of exosome-based therapies for diabetic wound healing. For example, Lv et al. reported that exosomes derived from human umbilical cord MSCs accelerate wound closure and promote angiogenesis in a diabetic rat model [55]. Similarly, Wang et al. demonstrated that exosomes derived from human amniotic epithelial cells promote re-epithelialization and collagen deposition in a diabetic mouse model [52]. However, these studies used exosomes derived from other cell types and did not specifically investigate the effects of fibroblast exosomes on diabetic wound healing.

Our study is unique in demonstrating the therapeutic efficacy of fibroblast exosomes for diabetic wounds. We showed that fibroblast exosome treatment not only accelerates wound closure but also improves the quality of healed skin in diabetic mice. Specifically, we demonstrated that fibroblast exosomes enhance epidermal barrier function, increase collagen deposition, and improve the biomechanical properties of healed diabetic skin. These findings are particularly significant given the impaired barrier function and reduced mechanical strength of diabetic skin [82].

Furthermore, our study provides mechanistic insights into how fibroblast exosomes promote diabetic wound healing. We showed that fibroblast exosomes reduce oxidative stress and activate the KEAP1/Nrf2 antioxidant pathway in diabetic wounds. These findings are consistent with previous reports demonstrating the importance of oxidative stress in the pathogenesis of diabetic wounds [83] and the potential therapeutic benefits of Nrf2 activation [64]. Our study extends these findings by showing that fibroblast exosomes can mitigate oxidative stress and promote Nrf2 activation in diabetic wounds.

Despite the promising therapeutic potential of fibroblast exosomes for diabetic wound healing, our study has several limitations that warrant further investigation. Clinically, T2DM has a significantly greater prevalence than T1DM and is typically associated with more complex comorbidities. Although our study employed a T1DM mouse model that may not fully recapitulate the intricate pathophysiological characteristics of diabetic wounds in humans, this selection was based on several key scientific considerations. First, our comparative analysis revealed that T1DM wounds exhibited similar patterns of healing impairment and cellular dysfunction to those of T2DM wounds, including delayed wound closure and compromised cellular responses. However, T1DM wounds presented more severe pathological manifestations, particularly with regards to fibroblast activation deficiency and exacerbated oxidative stress. Second, whereas T2DM stems from relative insulin deficiency leading to multiple metabolic disturbances, including hyperglycemia, elevated free fatty acids, and obesity, T1DM presents a more homogeneous metabolic profile dominated by hyperglycemia alone, thereby providing a more defined model for studying delayed wound healing [84,85]. With respect to ECM remodeling, T1DM primarily manifests as hyperglycemia-induced suppression of fibroblast proliferation and collagen synthesis, in contrast to T2DM, in which obesity-related inflammation predominantly drives ECM degradation. Therefore, T1DM is more suitable for investigating the role of fibroblast-derived exosomes in collagen deposition [86,87]. In terms of oxidative stress mechanisms, T1DM, characterized by absolute insulin deficiency, results in significantly elevated oxidative stress due to the loss of the intrinsic antioxidant capacity of insulin. In contrast, T2DM patients retain partial insulin secretory function, thereby preserving some components of the endogenous antioxidant defense system [88]. Critically, the suppression of the Nrf2 pathway represents a central defect in the antioxidant defense system in T1DM, making it particularly relevant to our experimental investigation [89,90]. Moving forward, we plan to expand our research to T2DM models and further validate our findings in large animal studies or clinical trials. Given the complexity of diabetic wound healing, we recognize that monotherapy alone may not fully address the multifactorial pathology. Therefore, combining exosome-based therapies with bioactive biomaterials represents a promising strategy, not only to increase exosome delivery and stability but also to achieve synergistic therapeutic effects through complementary mechanisms. This combined approach will be a key focus of our future investigations.

Although this study focused primarily on fibroblast-derived exosomes, a systematic comparison with other well-characterized exosome sources would allow for a more comprehensive evaluation of their therapeutic potential. Mesenchymal stem cell (MSC)-derived exosomes have been extensively investigated in wound healing research. For example, adipose-derived mesenchymal stem cells (ADSCs), which possess multipotent differentiation capacity (including osteogenic, chondrogenic, and adipogenic differentiation), secrete exosomes that effectively modulate inflammatory responses, promote collagen deposition, stimulate angiogenesis, and accelerate re-epithelialization during wound repair [91,92]. Similarly, exosomes from human umbilical cord mesenchymal stem cells (hUC-MSCs) and bone marrow-derived mesenchymal stem cells (BM-MSCs) function primarily during the proliferative phase of wound healing [93,94]. Additionally, immune cell-derived exosomes (e.g. macrophage-derived exosomes) mainly participate in wound repair through immunomodulatory mechanisms [95]. In contrast, the fibroblast exosomes investigated in this study demonstrate the following distinctive advantages: (i) enhanced type I/III collagen synthesis and deposition capacity, enabling direct regulation of extracellular matrix remodeling; (ii) more comprehensive wound healing promotion, beyond the tissue repair enhancement and antioxidative stress effects demonstrated in this study, our previous research revealed their ability to modulate macrophage polarization and effectively improve the inflammatory microenvironment of wounds [96]; and (iii) as native skin cells, fibroblast-derived exosomes exhibit superior tissue compatibility, significantly reducing tumorigenic risks and the incidence of immune rejection reactions. However, compared with MSCs, fibroblasts face limitations in their expansion capacity, necessitating further optimization of culture conditions and exosome isolation methods for large-scale production.

Ultracentrifugation is currently the gold standard for laboratory isolation of exosomes, offering the advantages of high purity and broad applicability. However, significant limitations exist when this method is applied in clinical practice. First, the powerful shear forces generated during ultracentrifugation may damage exosome membranes, compromising their bioactivity. Second, the equipment required is costly, and the process is time-consuming, making large-scale exosome production challenging. Additionally, storage and transportation requirements further limit their widespread clinical application. Future research should focus on (i) developing simpler, more efficient isolation techniques; (ii) identifying and characterizing key functional molecules within exosomes that could be synthetically reproduced to mimic vesicle effects; and (iii) employing bioengineering approaches using biomaterial-based exosome carriers to address storage/transportation challenges while simultaneously improving targeting specificity and reducing biological risk.

In conclusion, our study demonstrates the therapeutic potential of fibroblast exosomes for diabetic wound healing. We showed that fibroblast exosome treatment accelerated wound closure, promoted re-epithelialization, enhanced ECM deposition, and improved the quality of healed skin in a mouse model of type 1 diabetes. These effects are mediated, in part, by the activation of the KEAP1/Nrf2 antioxidant pathway and the reduction in oxidative stress. Our findings provide a strong rationale for further investigating fibroblast exosomes as a novel therapeutic strategy for diabetic wounds. However, additional studies are needed to validate our findings in more clinically relevant models, optimize delivery methods, and compare the efficacy of fibroblast exosomes to those of other therapeutic approaches. With further research and development, fibroblast exosomes may emerge as a promising cell-free therapy for the treatment of diabetic wounds and other chronic skin disorders.

## Conclusions

This study demonstrated that fibroblast exosomes significantly enhance wound healing by promoting the proliferation, migration, and differentiation of epidermal stem cells as well as improving granulation tissue formation and ECM deposition. In diabetic models, fibroblast exosomes accelerated wound closure and restored both the mechanical strength and epidermal barrier function of the skin. Mechanistic studies revealed that fibroblast exosomes activate the KEAP1/Nrf2 antioxidant pathway through miR-29a-3p, effectively reducing oxidative stress and protecting cells from ROS-induced damage. These findings highlight the therapeutic potential of fibroblast exosomes in both normal and diabetic wound healing, providing a theoretical foundation for the development of novel exosome-based therapeutic strategies.

## Supplementary Material

Figure_S1_tkaf035

Figure_S2_tkaf035

Figure_S3_tkaf035

Figure_S4_tkaf035

Figure_S5_tkaf035

Figure_S6_tkaf035

Figure_S7_tkaf035

Supplementary_materials_tkaf035

## Data Availability

The data that support the findings of this study are available from the corresponding author upon reasonable request.
