# Supplementary figures and images for: Fibroblast exosomes promote wound healing and improve the quality of healed skin via miR-29a-3p-mediated KEAP1/Nrf2 pathway activation
Source: Burns Trauma. 2025 May 17;13:tkaf035. doi: 10.1093/burnst/tkaf035 (PMC12554163; doi:10.1093/burnst/tkaf035)

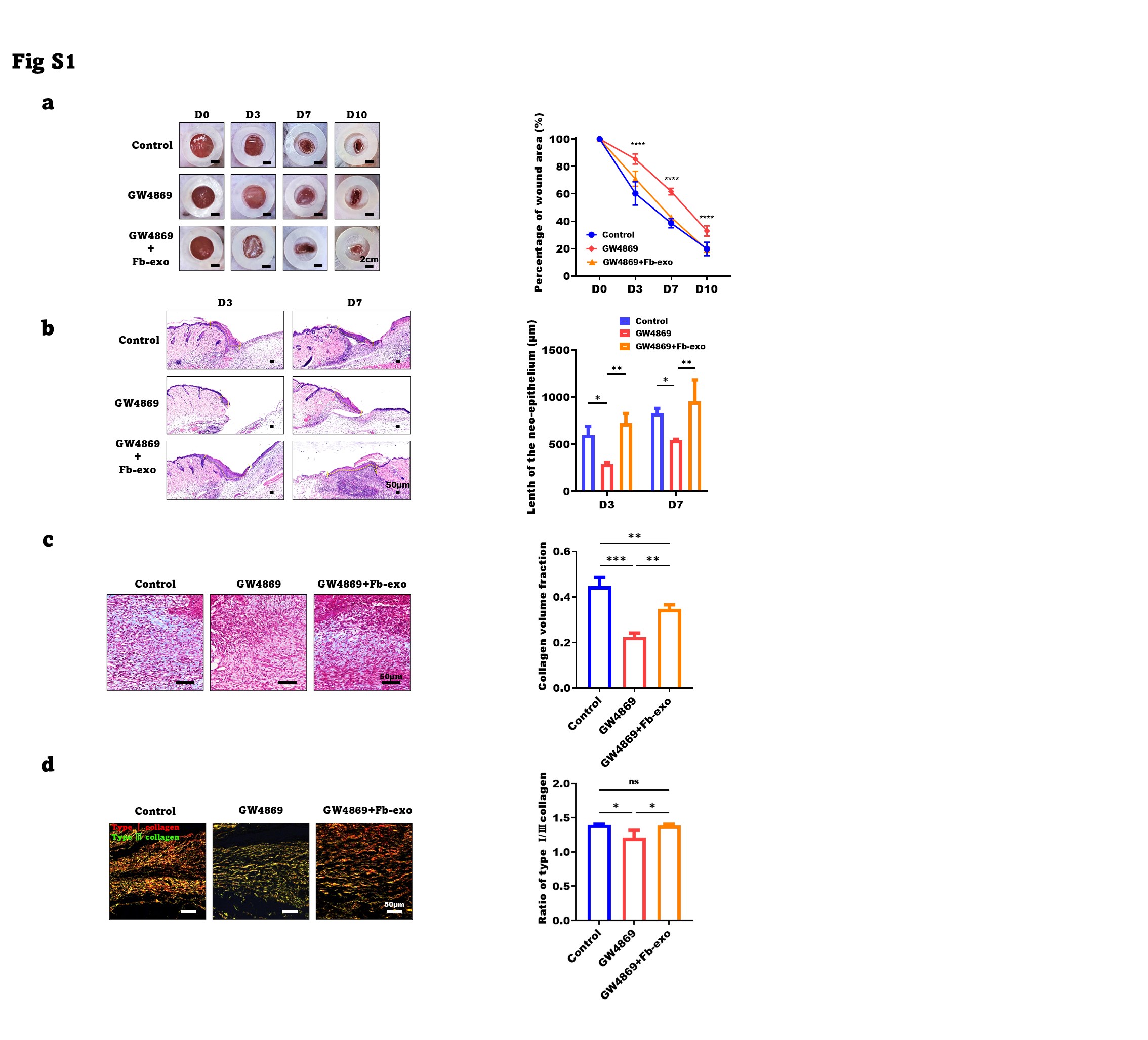

Supplement: Figure_S1_tkaf035 [file figure_s1_tkaf035.jpeg]

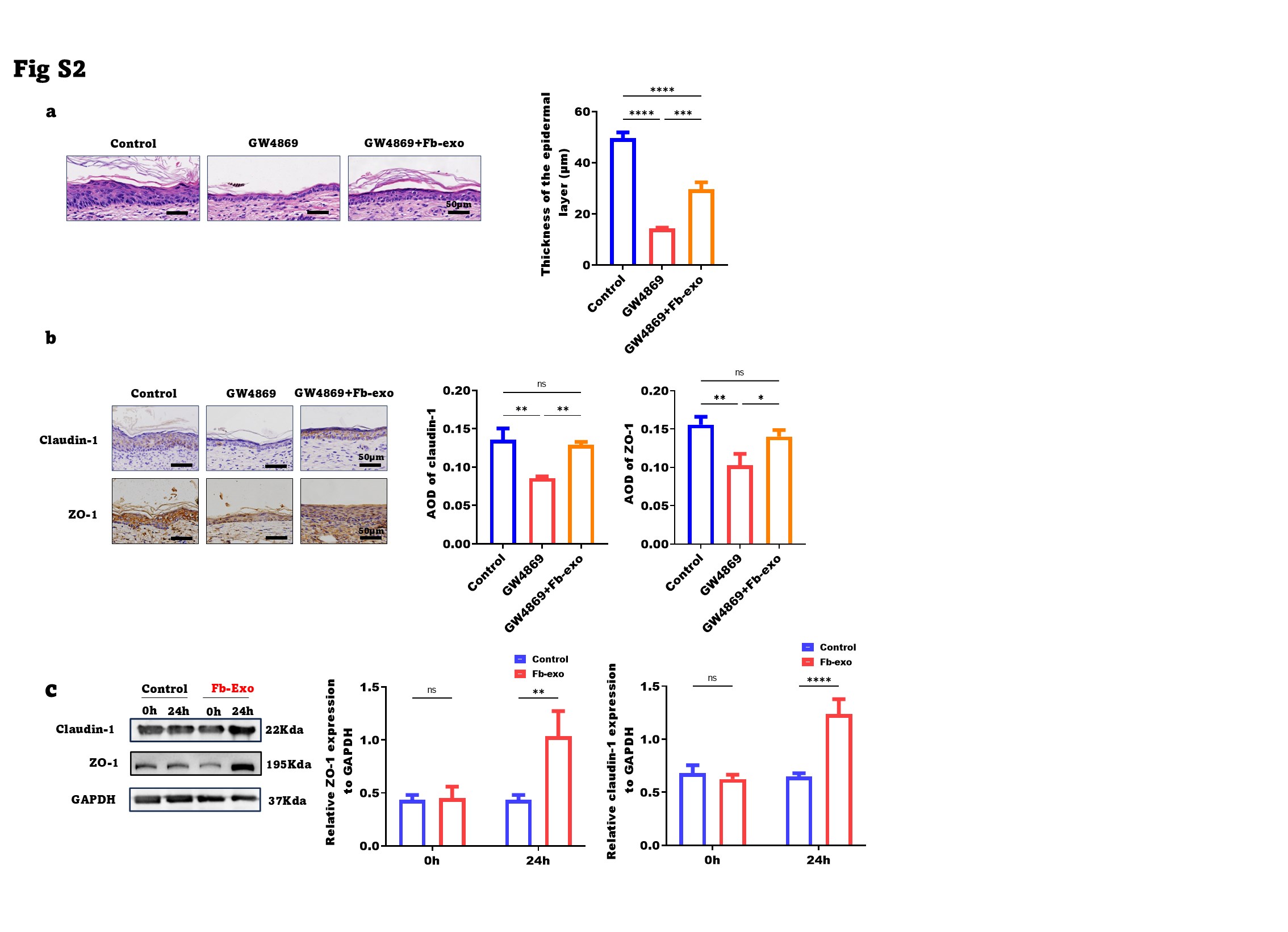

Supplement: Figure_S2_tkaf035 [file figure_s2_tkaf035.jpeg]

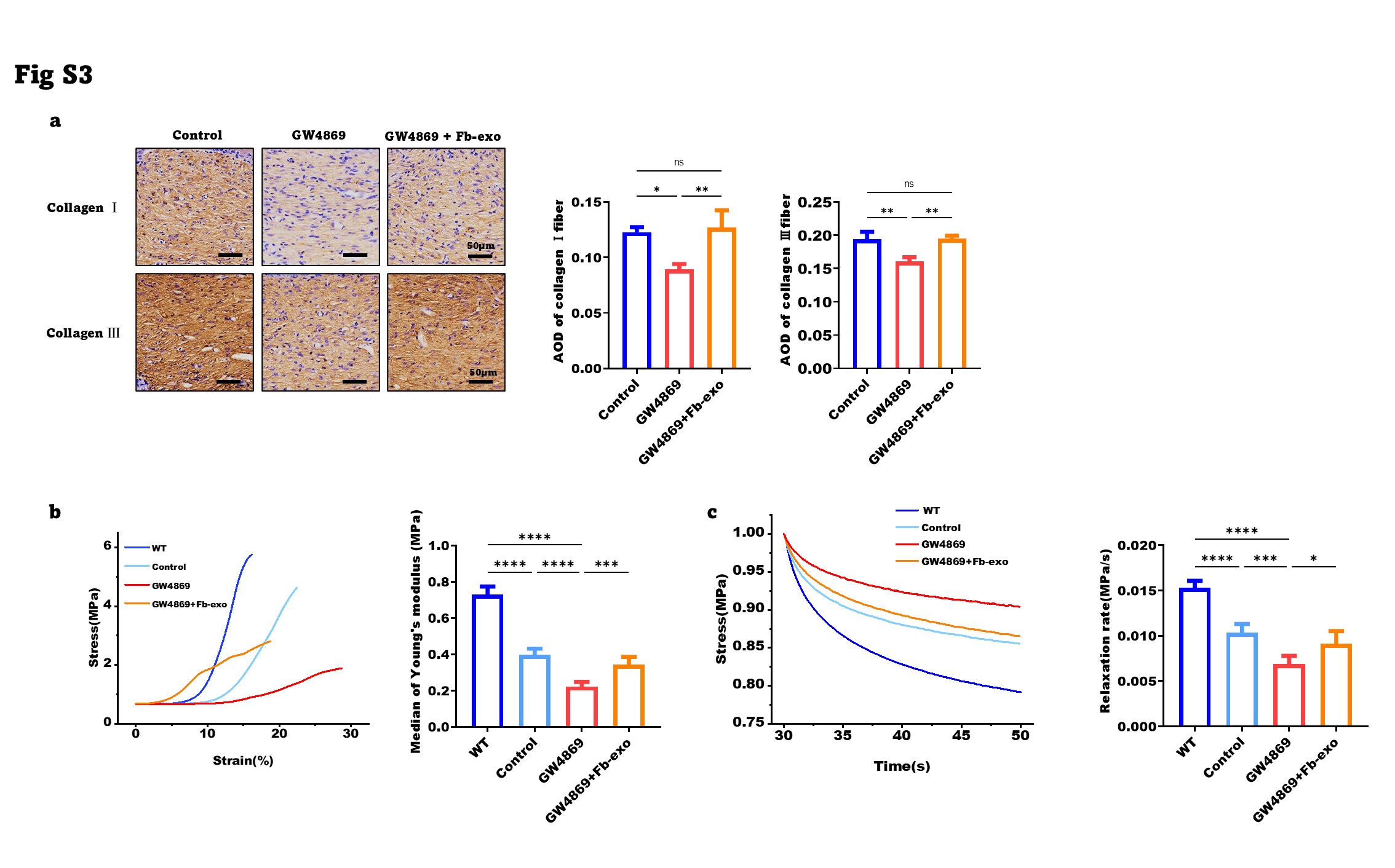

Supplement: Figure_S3_tkaf035 [file figure_s3_tkaf035.jpeg]

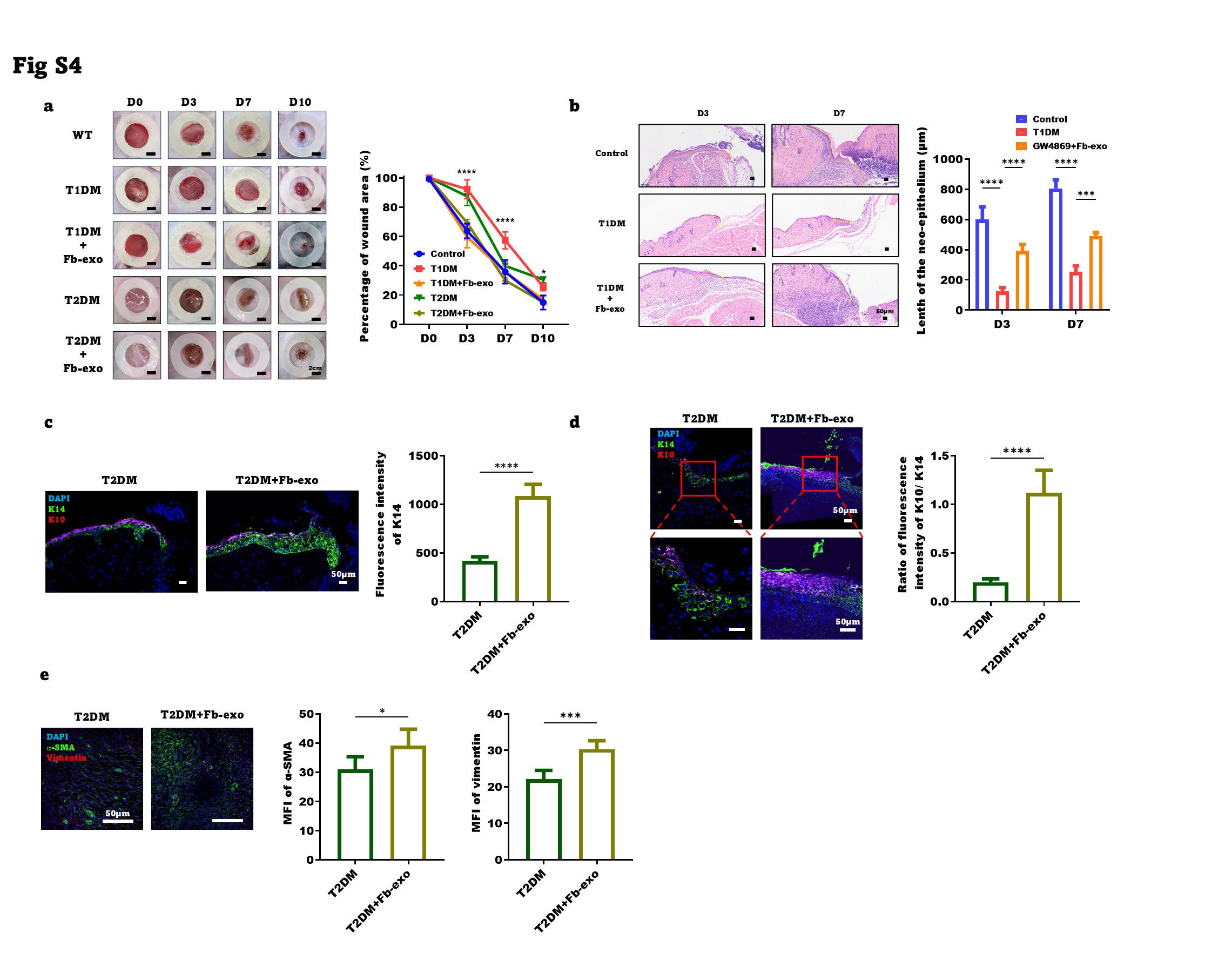

Supplement: Figure_S4_tkaf035 [file figure_s4_tkaf035.jpeg]

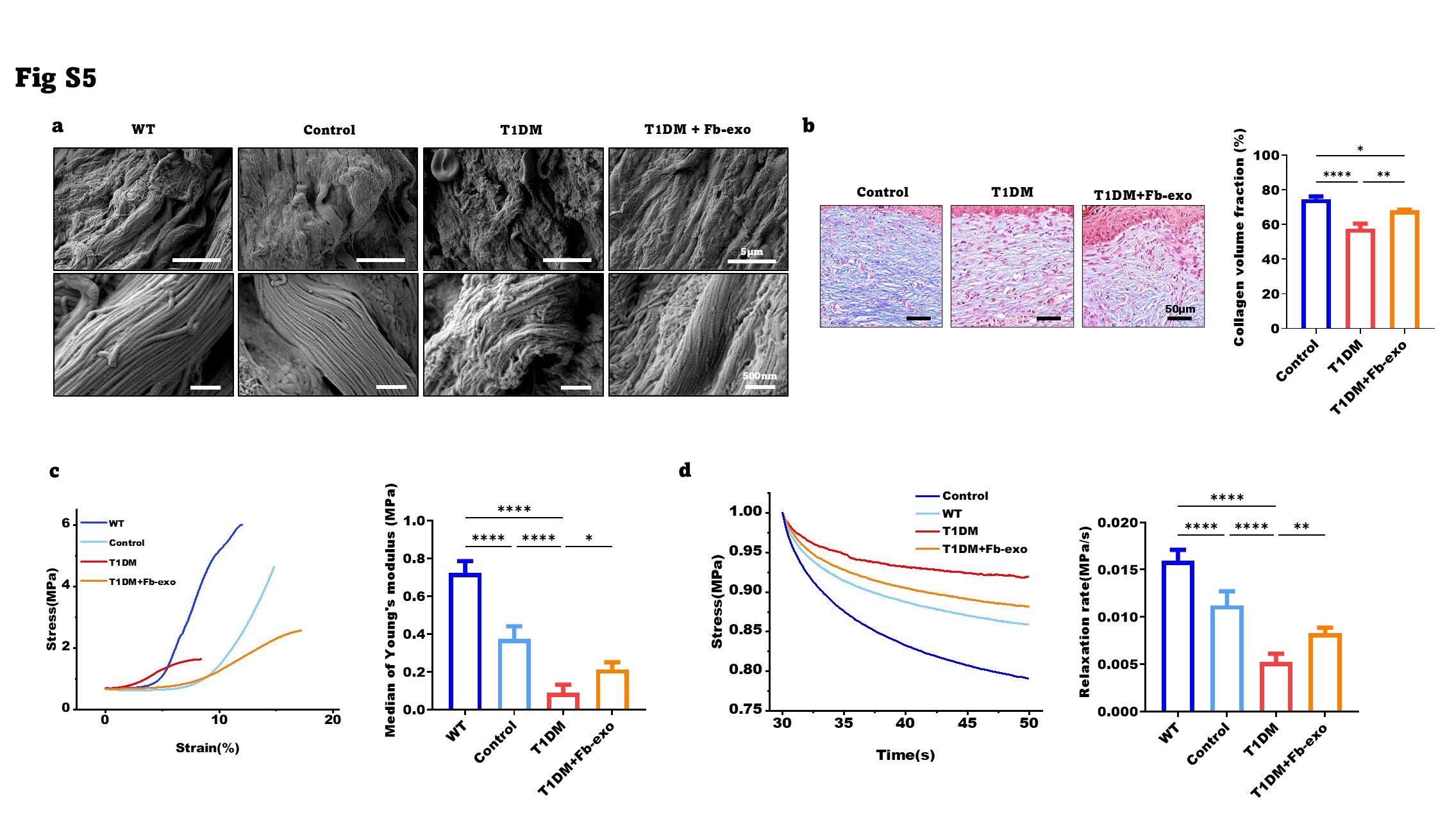

Supplement: Figure_S5_tkaf035 [file figure_s5_tkaf035.jpeg]

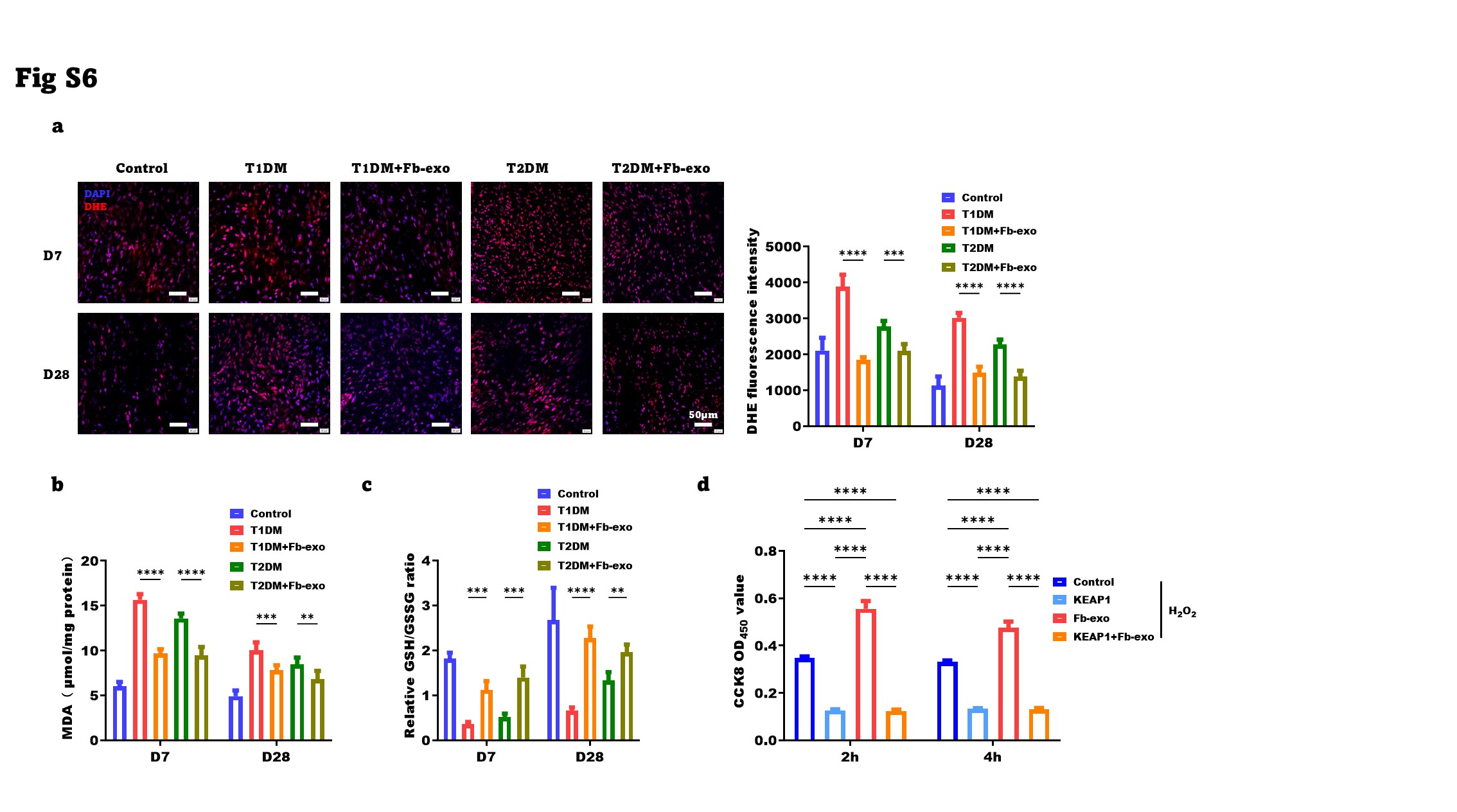

Supplement: Figure_S6_tkaf035 [file figure_s6_tkaf035.jpeg]

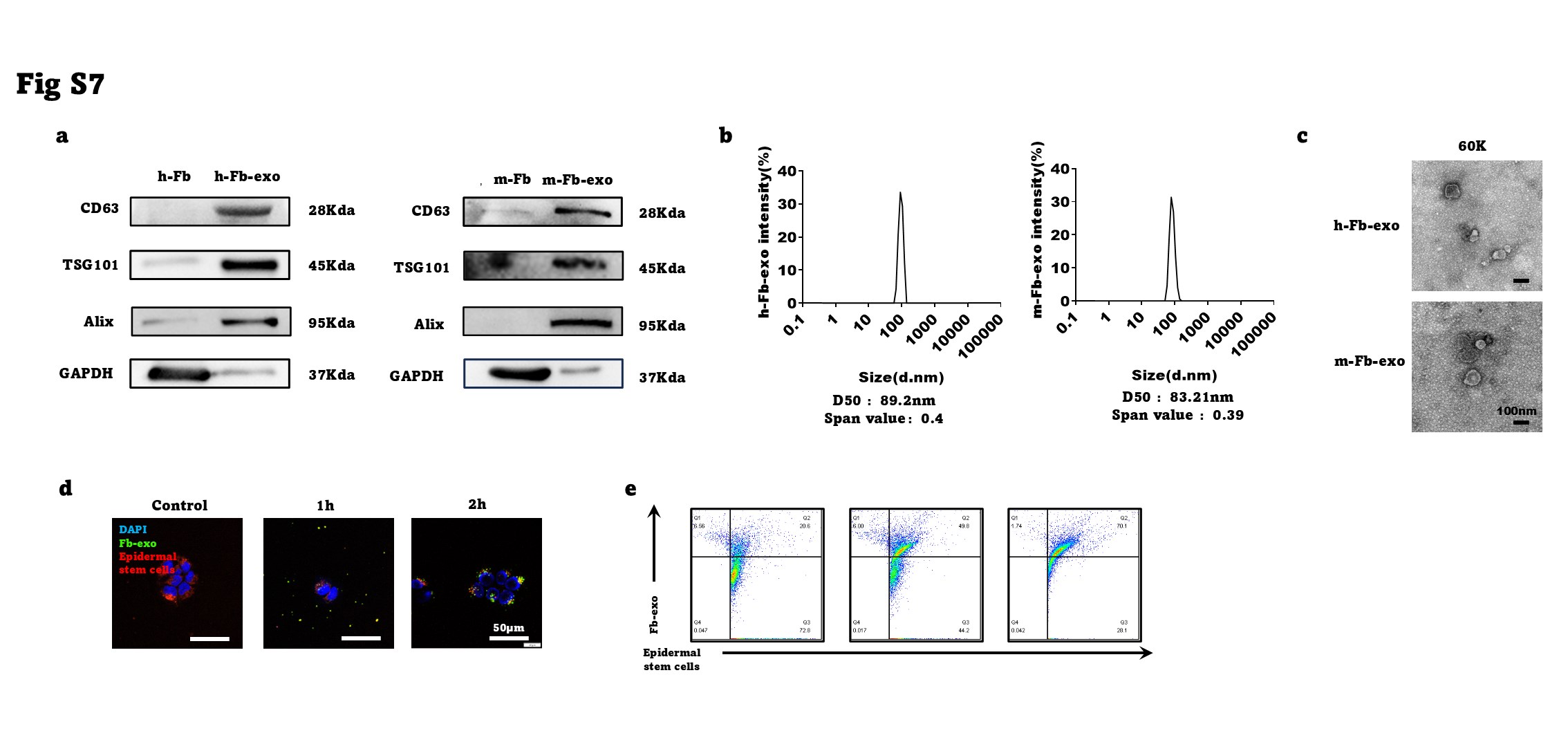

Supplement: Figure_S7_tkaf035 [file figure_s7_tkaf035.jpeg]
