# Supplementary material for: Fibroblast exosomes promote wound healing and improve the quality of healed skin via miR-29a-3p-mediated KEAP1/Nrf2 pathway activation
Source: Burns Trauma. 2025 May 17;13:tkaf035. doi: 10.1093/burnst/tkaf035 (PMC12554163; doi:10.1093/burnst/tkaf035)
Supplement: Supplementary_materials_tkaf035 [file supplementary_materials_tkaf035.docx]

**Supplementary materials**

**Figure S1.** **Fibroblast exosomes promote wound healing by enhancing re-epithelialization and collagen deposition**

1. Representative wound images and quantification of the wound area in the control, GW4869, and GW4869 + Fb-exo groups (scale bar=2 cm, n=5). (b) Detection of wound re-epithelialization by H&E staining. Representative images of the control, GW4869, and GW4869 + Fb-exo groups as well as the length of the new epithelial tongue (yellow line) and statistical analysis (scale bar=50 μm, n=3). (c) Determination of the collagen volume fraction of granulation tissue in the control, GW4869-treated, and GW4869 + Fb-exo groups via Masson’s trichrome staining (scale bar=50 μm, n=3). (d) Determination of the proportions of type I and type III collagen in the control, GW4869-treated, and GW4869 + Fb-exo groups by sirius red staining (scale bar=50 μm, n=3). Mean ± standard deviation (*p < 05; **p < 0.01; ***p < 0.001; ****p < 0.0001; ns not statistically significant).

**Figure S2.** **Fibroblast exosomes significantly improve the epidermal barrier function of the healed skin**

(a) Measurement of the thickness of the epidermal layer on day 28 post-wounding in the control, GW4869-treated, and GW4869 + Fb-exo groups and statistical analysis (scale bar=50 μm, n=3). (b) Expression levels of the tight junction proteins claudin-1 and Zo-1 in the healed wound tissue of the control, GW4869-treated, and GW4869 + Fb-exo groups, showing representative IHC images and statistical analysis (scale bar=50 μm, n=3). (c) Expression levels of tight junction proteins after stimulation with fibroblast exosomes for 24 h, as shown by representative WB images and statistical analysis. Mean ± standard deviation (*p < 05; **p < 0.01; ***p < 0.001; ****p < 0.0001; ns not statistically significant). *GAPDH* glyceraldehyde-3-phosphate dehydrogenase, *ZO-1* Zonula Occludens-1, *AOD* average optical density.

**Figure S3.** **Fibroblast exosomes significantly improve the collagen composition and long-term mechanical properties of healed skin**

(a) The expression levels of type I and type III collagen in the healed skin of the control, GW4869, and GW4869 + Fb-exo groups were determined via IHC (scale bar=50 μm, n=3). (b) Representative stress‒strain diagram of the healed skin and statistical analysis of the Young's modulus of the control, GW4869, and GW4869+Fb-exo groups (n=5). (c) Representative diagram of the stress relaxation curve of the healed skin and statistical analysis of the relaxation rates of the control, GW4869, and GW4869+Fb-exo groups (n=5). Mean ± standard deviation (*p < 0.05; **p < 0.01; ***p < 0.001; ****p < 0.0001; ns not statistically significant). *WT* wild type, *AOD* average optical density.

**Figure S4. Fibroblast exosomes promote re-epithelialization of diabetic wounds and improve fibroblast activation**

(a) Representative wound images and quantification of the wound area in the control, T1DM, T1DM+Fb-exo, T2DM, and T2DM+Fb-exo groups (scale bar=2 cm, n=5). (b) Hematoxylin and eosin (H&E) staining was used to detect wound re-epithelialization. Representative images of the control, T1DM and T1DM+Fb-exo groups, as well as the length of the new epithelial tongue (yellow line) and statistics (scale bar=50 μm, n=3). (c) Representative images of the ratio of K10 to K14 fluorescence on day 7 post-wounding in the T2DM and T2DM+Fb-exo groups. Statistical analysis of the K14 fluorescence intensity (scale bar=50 μm, n=5). (d) Representative images of the ratio of K10 terminal epidermal cells to K14 progenitor cells in the T2DM and T2DM+Fb-exo groups. Statistical analysis of the ratio of K10 and K14 fluorescence intensities (scale bar=50 μm, n=5). (e) Immunofluorescence staining of vimentin and α-SMA on day 7 after skin wound creation in the T2DM and T2DM+Fb-exo groups is shown, and statistical analysis was performed (scale bar=50 μm, n=5). Mean ± standard deviation (*p < 0.05; **p < 0.01; ***p < 0.001; ****p < 0.0001; ns not statistically significant). *T1DM* type 1 diabetes mellitus*, T2DM* type 2 diabetes mellitus*, DAPI* 4',6-Diamidino-2-Phenylindole, *K10* Keratin 10, *K14* Keratin 14, *MFI* mean fluorescence intensity, *α-SMA* α-smooth muscle actin.

**Figure S5. Fibroblast exosomes significantly improve ECM organization and long-term mechanical properties of healed skin in diabetic wounds**

(a) Scanning electron microscopy images of the morphology of collagen fibers in the wounds of mice in the control, T1DM and T1DM+Fb-exo groups on day 28 (scale bar = 5 μm, 500 nm; n=5). (b) Masson staining was used to determine the collagen volume fraction in the granulation tissue of the healed wounds of mice in the control, T1DM and T1DM+Fb-exo groups (scale bar=50 μm, n=3). (c) Representative stress‒strain diagram of the healed skin and statistical analysis of the Young's modulus of the control, T1DM, and T1DM+Fb-exo groups (n=5). (d) Representative diagram of the stress relaxation curve of the healed skin and statistical analysis of the relaxation rates of the control, T1DM, and T1DM+Fb-exo groups (n=5). Mean ± standard deviation (*p < 0.05; **p < 0.01; ***p < 0.001; ****p < 0.0001; ns not statistically significant). *WT* wild type, *T1DM* type 1 diabetes mellitus.

**Figure S6. Fibroblast exosomes improve diabetic wound healing quality by reducing oxidative stress damage**

(a) Representative images of DHE staining and statistical analysis of the fluorescence intensity of fresh frozen tissue sections from wounds in the control, T1DM, T1DM+Fb-exo, T2DM, and T2DM+Fb-exo groups on days 7 and 28 post-wounding (scale bar=50 μm, n=5). (b) Statistical analysis of the MDA concentration in wound tissue from the control, T1DM, T1DM+Fb-exo, T2DM, and T2DM+Fb-exo groups on days 7 and 28 after wounding (n=5). (c) Statistical analysis of the ratio of GSH/GSSG in wound tissue in the control, T1DM, T1DM+Fb-exo, T2DM, and T2DM+Fb-exo groups on days 7 and 28 after wounding (n=5). (d) Statistical analysis of cell viability after epidermal stem cells were stimulated with fibroblast exosomes and recombinant KEAP1 proteins under oxidative stress conditions. Mean ± standard deviation (*p < 05; **p < 0.01; ***p < 0.001; ****p < 0.0001; ns not statistically significant). *T1DM* type 1 diabetes mellitus*, T2DM* type 2 diabetes mellitus*, DAPI* 4',6-Diamidino-2-Phenylindole, *DHE* dihydroethidium, *MDA* malondialdehyde, *GSH* Glutathione, *GSSG* Glutathione Disulfide, *CCK8* cell counting kit-8.

**Figure S7.** **Characterization of fibroblast exosomes**

(a) Representative images of protein immunoblot bands of CD63, TSG101, Alix, and GAPDH in human and mouse fibroblast exosomes (n=3). (b) Representative results of particle size analysis of human and mouse fibroblast exosomes (n=3). (c) Representative transmission electron microscopy images of human and mouse fibroblast exosomes (scale bar=100 nm, n=5). (d) Representative fluorescence images of epidermal stem cells phagocytosing fibroblast exosomes at 1 h and 2 h post-stimulation (scale bar=50 μm, n=3). (e) Representative flow cytometry plots of EpSCs phagocytosing fibroblast exosomes at 1 h and 2 h post-stimulation (n=3). *TSG101* tumor susceptibility Gene 101, *Alix* apoptosis-linked gene 2-interacting protein X, GAPDH glyceraldehyde-3-phosphate dehydrogenase, *DAPI* 4',6-Diamidino-2-Phenylindole.
